# Comprehensive proteomic analysis of autophagosomes derived from *Leishmania*-infected macrophages

**DOI:** 10.1371/journal.pone.0284026

**Published:** 2023-04-07

**Authors:** Devki Nandan, Eleanor Chen, Fabian Chang, Kyung-Mee Moon, Leonard J. Foster, Neil Reiner

**Affiliations:** 1 Division of Infectious Diseases, Department of Medicine, University of British Columbia, Vancouver, British Columbia, Canada; 2 Department of Biochemistry and Molecular Biology, University of British Columbia, Vancouver, British Columbia, Canada; Institut national de la recherche scientifique, CANADA

## Abstract

Recently, autophagy has been implicated as a host defense mechanism against intracellular pathogens. On the other hand, certain intracellular pathogens such as *Leishmania* can manipulate the host’s autophagy to promote their survival. Our recent findings regarding the regulation of autophagy by *Leishmania donovani* indicate that this pathogen induces non-classical autophagy in infected macrophages, independent of regulation by the mammalian target of rapamycin complex 1. This suggests the fine-tuning of autophagy to optimally promote parasite survival, possibly by the sequestration or modulation of specific autophagosome-associated proteins. To investigate how *Leishmania* potentially manipulates the composition of host-cell autophagosomes, we undertook a quantitative proteomic study of the human monocytic cell line THP-1 following infection with *L*. *donovani*. We used stable isotope labeling by amino acid in cell culture and liquid chromatography-tandem mass spectrometry to compare expression profiles between autophagosomes isolated from THP-1 cells infected with *L*. *donovani* or treated with known autophagy inducers. Selected proteomic results were validated by Western blotting. In this study, we showed that *L*. *donovani* modulates the composition of macrophage autophagosomes during infection when compared to autophagosomes induced by either rapamycin (selective autophagy) or starvation (non-selective autophagy). Among 1787 proteins detected in *Leishmania*-induced autophagosomes, 146 were significantly modulated compared to the proteome of rapamycin-induced autophagosomes, while 57 were significantly modulated compared to starvation-induced autophagosomes. Strikingly, 23 *Leishmania* proteins were also detected in the proteome of *Leishmania*-induced autophagosomes. Together, our data provide the first comprehensive insight into the proteome dynamics of host autophagosomes in response to *Leishmania* infection and demonstrate the complex relations between the host and pathogen at the molecular level. A comprehensive analysis of the *Leishmania*-induced autophagosome proteome will be instrumental in the advancement of understanding leishmaniasis.

## Introduction

Leishmaniasis is a vector-borne disease caused by an intracellular protozoan parasite belonging to the genus *Leishmania*. Depending on the *Leishmania* species and the host immune response, leishmaniasis may present clinically as a cutaneous, mucocutaneous, or visceral disease. In humans, visceral leishmaniasis is mainly caused by *Leishmania donovani* and it represents the most severe form of the disease [1, 2]. Although anti-leishmanial therapy has improved over time, it still has limited efficacy for leishmaniasis treatment and is highly toxic to healthy cells. Thus, identifying novel therapeutic strategies is an important goal in leishmaniasis research. In order to develop these therapies, a comprehensive understanding of host-pathogen interactions is required. It is becoming increasingly clear that *Leishmania* employs various clever tactics to survive in the hostile environment of host macrophages [3]. Recently, it has been shown that *Leishmania* takes advantage of host-macrophage autophagy to promote its survival [4, 5].

Macroautophagy (hereafter referred to as autophagy) is a conserved lysosomal catabolic pathway that degrades damaged organelles, misfolded protein aggregates, and invading pathogenic microbes. In autophagy, cellular contents are delivered by double-membrane vesicles, called autophagosomes, to lysosomes for destruction in either a selective or non-selective manner [6]. Non-selective autophagy is a bulk degradative process, mainly induced by cellular stressors such as amino acid and ATP deprivation [7]. Alternatively, selective autophagy involves the recycling of specific organelles such as mitochondria, peroxisomes, and proteasomes. The ability to selectively clear organelles, intracellular pathogens, and cellular components via selective autophagy makes it a critical process for the homeostasis of eukaryotic cells. Conversely, dysfunctional autophagy contributes to many diseases, including cancer, and promotes the survival of intracellular pathogens [8]. Therefore, modulating autophagy for the treatment of conditions such as cancer and intracellular pathogenic diseases is a promising therapeutic approach currently under intense investigation.

Autophagy is a complex process involving the interplay between many proteins. Specifically, autophagy relies on a core set of autophagy-related (ATG) proteins [9]. Among these proteins, the lipid conjugated protein marker, microtubule-associated protein 1 light chain 3b (LC3-II/ATG8) associates with the autophagosomal double membrane and has been used extensively as an indicator of autophagy in a wide variety of cells and tissues [10]. The main regulatory pathway of classical autophagy is dependent on the mammalian target of rapamycin complex 1(mTORC1) and involves the PI3K/AKT/mTORC1 signaling pathway [11–13]. In addition, mTORC1 has been implicated in controlling autophagosome maturation in nutrient-deprived conditions [11]. However, there is compelling evidence that shows that autophagy can also be regulated via mTORC1-independent pathways [7, 14, 15].

Recently, autophagy has been implicated in the defense against intracellular pathogens [16]. Notably, autophagy in macrophages attenuates the survival of numerous pathogens such as *Mycobacterium tuberculosis*, *Shigella flexneri*, *and Listeria monocytogenes* [16]. On the other hand, certain intracellular pathogens such as *T*. *gondii* [17], Hepatitis C virus [18], and *Coxiella burnetiid* [19] appear to have evolved to regulate host autophagy to their advantage. Furthermore, several species of *Leishmania* have been observed to induce macrophage autophagy [5, 20–23]. Our recent findings indicate that *L*. *donovani* uses dual strategies to exert countervailing effects on host autophagy. More specifically, the parasite actively inhibits mTORC1-dependent host-cell autophagy during infection through the sustained activation of the PI3K/Akt pathway [4]. Conversely, *Leishmania* promotes mTORC1-independent autophagy at later stages of infection in the background of enhanced mTOR activity [4]. In addition, downregulation of host macrophage Atg5 or Atg9A (essential autophagy proteins) resulted in a marked decrease in *Leishmania* survival, directly linking host autophagy to *Leishmania* pathogenesis [4].

However, the role of autophagy in promoting the survival of *Leishmania* in infected cells in the background of sustained host PI3K/AKT/mTORC1 pathway induction has yet to be investigated. Therefore, we sought to identify autophagosome-associated proteins isolated from *L*. *donovani*-infected host cells by quantitative proteomic analysis. For this study, we used differentiated THP-1 cells as model phagocytic cells, which have been extensively used to study experimental leishmaniasis [4, 24–26]. For comparison, proteomic analysis was performed on autophagosomes isolated from host cells treated with two extensively studied autophagic stimuli: amino acid deprivation (non-selective autophagy) and rapamycin (selective autophagy). The data presented here provide a quantitative proteomic characterization of *Leishmania*-induced autophagosome-associated proteins, where, strikingly, *Leishmania* proteins were also detected. In fact, to the best of our knowledge, this the first report to comprehensively identify a repertoire of autophagosome-associated proteins isolated from host macrophages infected with *Leishmania*, or with any intracellular pathogens for that matter. Interestingly, there are similarities between the protein compositions of *Leishmania*- and starvation-induced autophagosomes. Additionally, we discuss resulting insights into mTOR-independent host macrophage autophagy induced during *Leishmania* infection.

## Materials and methods

### THP-1 cell culture and stable isotope labeling by amino acid in cell culture (SILAC)

THP-1 cells, obtained from ATCC (TIB-202TM), were cultured at 37°C with 5% CO_2_ in RPMI-1640 media (Gibco) containing 10% heat-inactivated fetal calf serum (Gibco), 10 mM HEPES (MilliporeSigma), 100 units/mL penicillin-streptomycin (MilliporeSigma), and 2 mM L-glutamine (Gibco). For SILAC, THP-1 cells were grown in L-glutamine, L-arginine, and L-lysine deficient RPMI-1640 (Caisson Labs), supplemented with 100 units/mL penicillin-streptomycin (MilliporeSigma), 2 mM L-glutamine (MilliporeSigma), 7% dialyzed fetal bovine serum (Gibco) and labeled with either L-lysine and L-arginine (Lys0, Arg0), L-lysine-^2^H_4_ and L-arginine-^13^C_6_ (Lys4, Arg6), or L-lysine ^13^C_6_-^15^N_2_ and L-arginine ^13^C_6_-^15^N_4_ (Lys8, Arg10). Stable isotope-labeled amino acids were purchased from Cambridge Isotope Laboratories (Andover, MA, USA). For experiments, THP-1 cells were differentiated with 10 ng/mL of phorbol 12-myristate 13-acetate (PMA) (MilliporeSigma) for 16–18 h. The differentiated THP-1 cells (dTHP-1) were rested in fresh media for 6 h prior to treatments.

### *L*. *donovani* culture and infection

Sudan strain S2 promastigotes were cultured at 26°C, in M199 media (MilliporeSigma) with 7% heat-inactivated fetal calf serum (Gibco), 20 mM HEPES (MilliporeSigma), 100 units/mL penicillin-streptomycin (MilliporeSigma), 100 μM adenosine (MilliporeSigma), 2 mM L-glutamine (Gibco), 3 μg/mL hemin (MilliporeSigma), and 10 μg/mL folic acid (MilliporeSigma). Every third day, promastigotes were passaged (1:10) into fresh medium. For infections, dTHP-1 cells were incubated with stationary phase *Leishmania* promastigotes at an MOI (Multiplicity of Infection) of 20:1 for 24 hours. As a phagocytic control, dTHP-1 cells were treated with 2.0 micron PolyBeads^®^ (Polysciences Inc.) at an MOI of 5:1, 10:1 and 20:1. Following 24 hours of infection, the cells were thoroughly washed to remove uninternalized parasites or beads and immediately collected for further processing and analysis. For experiments, we used cells with an infection rate of 70% or higher and an intracellular parasite count ranging from 2–10 per cell. To maintain the virulence of *Leishmania*, parasites were routinely isolated from *Leishmania*-infected Syrian Golden hamsters.

### Determination of intracellular parasite infection rate and burden

To determine parasite infection rate, dTHP-1 cells were infected for 24 hours, washed, and then fixed onto coverslips using 2% paraformaldehyde in PBS for 15 min. The coverslips were mounted onto glass slides using Prolong^™^ Diamond Antifade Mountant with DAPI (Life Technologies Inc.). Infected cells were imaged using the Zeiss Axioplan 2 imaging microscope and the percentage of infected macrophages was calculated. For parasite burden, the number of parasites per 100 macrophages was determined (S1 Fig).

### Autophagosome purification

For autophagy induction, dTHP-1 cells were either treated with 12.5 μg/mL rapamycin (Sigma), infected with *L*. *donovani*, or amino acid-starved in HBSS (Gibco) and treated with 100 nM Bafilomycin A1 (Sigma). The autophagosomes were purified as previously described [27, 28] with some modifications. 30×10^6^ dTHP-1 cells from each experimental condition were washed and harvested in 5 mL homogenization medium (HM: 0.25 M sucrose, 1 mM EDTA, 20mM Tris-HCl, pH 7.4, protease inhibitor cocktail (cOmplete^™^ Mini EDTA-free protease inhibitor cocktail), 5 μg/mL aprotinin, 5μg/mL leupeptin, 2 mM PMSF; all reagents are from MilliporeSigma) by gentle scraping. Cells from the three treatments were pooled, pelleted at 250 × g, 4°C, for 5 min, and then resuspended in 600 μL HM. The resuspended cells were fractionated by sequential centrifugation at 1000, then 3000 x g for 10 min at 4°C, where pellets were discarded. The supernatant was then centrifuged at 17 000 × g, at 4°C for 15 min. The resulting pellet was resuspended in 1 mL HM, split in half, and loaded on top of two iodixanol (Stem Cell) gradients composed of five 800 μL iodixanol fractions (5%, 10%, 16%, 24%, and 30% iodixanol). Using the Sorvall MTX 150 micro-ultracentrifuge with the S110AT-0088 fixed angle rotor, the sample was separated on the gradient at 100 000 × g, 4°C, for 17 h. Six fractions of 800 μL were collected and proteins from each fraction were concentrated via deoxycholate (Sigma)—trichloroacetic acid (Merck) (DOC-TCA) precipitation. In brief, samples were incubated with 0.015% DOC for 10 min at room temperature followed with 10% TCA for 30 min on ice, and then centrifuged at 13 000 × g for 15 min at 4°C. Iodixanol was removed via two washes with 10% TCA. TCA and DOC were removed via two acetone washes and proteins were resuspended in 2% SDS in 10 mM Tris-HCl, pH 7.5. The 16% iodixanol fraction from three biological replicates were used for liquid chromatography-tandem mass spectrometry (n = 3).

### Liquid chromatography-tandem mass spectrometry (LC/MS-MS) and protein identification

Equal amounts of protein from each replicate were reduced and alkylated [29], and run on 10% SDS-PAGE. The entire lane was split into 5 fractions, and digested in gel with MS-grade trypsin (Promega) [30] and the resulting peptides were cleaned on C-18 Stop And Go Extraction (STAGE) tips [31] using 40% (v/v) acetonitrile in 0.1% (v/v) formic acid as the elution buffer. Peptides were analyzed on NanoDrop One (Thermo Fisher—A205, scopes) to load approximately 300 ng of peptides on Impact II Qtof (Bruker Daltonics) coupled to easy nLC 1200 (Thermo Scientific). Ionopticks’s Aurora series 25 cm x 75 μm C18 1.6μm analytical column heated to 50°C was used for a 90 min separation with acquisition settings as described [32]. Acquired data were searched on MaxQuant version 1.6.17.0 [33] against UniProt’s human sequences (UP000005640), *Leishmania donovani* BPK282A1, and common contaminant sequences. SILAC labels of medium arginine (^13^C_6_), medium lysine (^2^H_4_), heavy arginine (^13^C_6_, ^15^N_4_), and heavy lysine (^13^C_6_, ^15^N_2_) were set for quantitation enabling “iBAQ”, “requantify”, and “match-between-runs” options. The data were filtered for 1% false discovery at protein, peptide, and PSM levels. For each LC-MS run, peptide ratios were normalized in MaxQuant so that the median of their logarithms are zero to control for unequal protein loading [34]. The mass spectrometry proteomics data have been deposited to the ProteomeXchange Consortium via the PRIDE [35] partner repository with the dataset identifier PXD037057. The reviewer account for the PRIDE data has the username reviewer_pxd037057@ebi.ac.uk and the password 2zda4ZAt.

### Antibodies

Primary antibodies for LC3-II, p62/SQSTM1 (Sequestosome-1), and β-actin were obtained from Cell Signaling Technology. Primary antibodies for Lyn and Annexin V were obtained from Santa Cruz Biotechnologies. Peroxidase-conjugated affinity-purified anti-rabbit and anti-mouse IgG (H&L) (goat) secondary antibodies were obtained from Calbiochem. Polyclonal antisera against *Leishmania* elongation factor-1 alpha (*Ld*-EF-1α) were produced as previously described [36]. Antibodies against *Leishmania* fructose-bisphosphate aldolase (*Ld*-aldolase) were commercially produced by Genemed Synthesis Inc by immunizing rabbits with *Leishmania* aldolase-specific peptide (CMAQLGKYQRAHDNAS).

### Western blotting

To generate whole cell lysate, dTHP-1 cells or *L*. *donovani* promastigotes were washed and lysed in ice-cold lysis buffer (20 mM Tris-HCl, pH 6.8, 1% Triton X-100, 1 mM EDTA, 0.15 M NaCl, 1 mM sodium orthovanadate, 5 mM NaF, 5 μg/mL aprotinin, 5 μg/mL leupeptin, and 2mM PMSF; all reagents are from MilliporeSigma). Whole cell lysates or autophagosomal fractions were separated using Tris/Tricine or Tris/Glycine SDS-PAGE and transferred onto polyvinylidene difluoride or nitrocellulose membranes respectively. For LC3-II, membranes were blocked in a mixture of 5% milk and 3% BSA in 1X tris-buffered saline with 0.1% Tween-20 (TBS-T) and probed with α-LC3-II antibodies overnight at 4°C. For p62/SQSTM-1, Lyn, Annexin A5, GAPDH, and *Ld*-aldolase, membranes were blocked with 5% milk in TBS-T and probed with their respective primary antibodies for 1 h at room temperature or overnight at 4°C (GAPDH). For *Ld*-EF-1α, autophagosomal fractions were dotted directly onto a nitrocellulose membrane, blocked with 5% milk in TBS-T and incubated with α-EF-1α antibodies for 1 h at room temperature. The membranes were incubated with the appropriate secondary antibodies and protein bands were developed using ECL SuperSignal^™^ West Pico PLUS (ThermoFisher Scientific).

### Quantitative proteome analysis

The MaxQuant output file was analyzed using Perseus (version 1.6.15.0). Proteins from the reverse database, proteins only identified by site, and potential contaminants were filtered out. Proteins present in at least two replicates with a non-zero intensity were considered to be reliably detected within the treatment condition. For comparisons between treatments, the proteins that were present in all three replicates with a minimum of two peptides were used for further analysis. The SILAC ratios of these proteins were then log2 transformed. Statistical analysis was performed using a two-tailed, one-sample t-test and was compared against a value of 0. Proteins that were modulated by at least 75% with p < 0.05 were considered significantly altered. The data was uploaded to R (v4.2.0) using the RStudio (v2022.07.1) interface and a volcano plot was generated using the ggplot2 (v3.3.6) and dplyr (v1.0.9) packages.

### Gene ontology and cellular localization analysis

Gene Ontology (GO) analysis was performed using the GOTermMapper tool by Princeton University (https://go.princeton.edu/cgi-bin/GOTermMapper). Protein IDs of all *Leishmania*-infected autophagosome-associated proteins, as well as all modulated proteins, were input into the GOTermMapper with the following settings: Ontology Aspects set to Process, Organism set to Homo Sapiens (GOA@EBI + Ensembl), and Ontology set to Generic slim. To summarize and visualize the findings, the number of up-modulated and down-modulated proteins belonging to each GO category was graphed using ggplot2 (v3.3.6), dplyr (v1.0.9), and forcats (v0.5.1) in RStudio (v2022.07.1). For brevity, GO categories with less than 10% of the number of proteins in the largest categories were omitted from the graph and similar GO categories were grouped together as noted for improved figure clarity (S1 Table).

For cellular localization analysis, protein IDs of all *Leishmania*-infected autophagosome-associated proteins were input into UniProt’s ID mapping tool (From UniProtKB AC/ID database to UniProtKB database). The generated list was visualized using ggplot2 (v3.3.6) and forcats (v0.5.1) in RStudio (v2022.07.1). For brevity, proteins with an organelle membrane or sub-organelle structure localization were relabeled as the corresponding organelle. Sub-organelle structures were determined using UniProt’s subcellular location descriptions. All duplicate entries were removed and categories with less than 10% of the number of proteins in the largest category were omitted from the graph to improve figure clarity.

## Results

### Quantitative mass spectrometric analysis of autophagosomes

**Fig 1:**
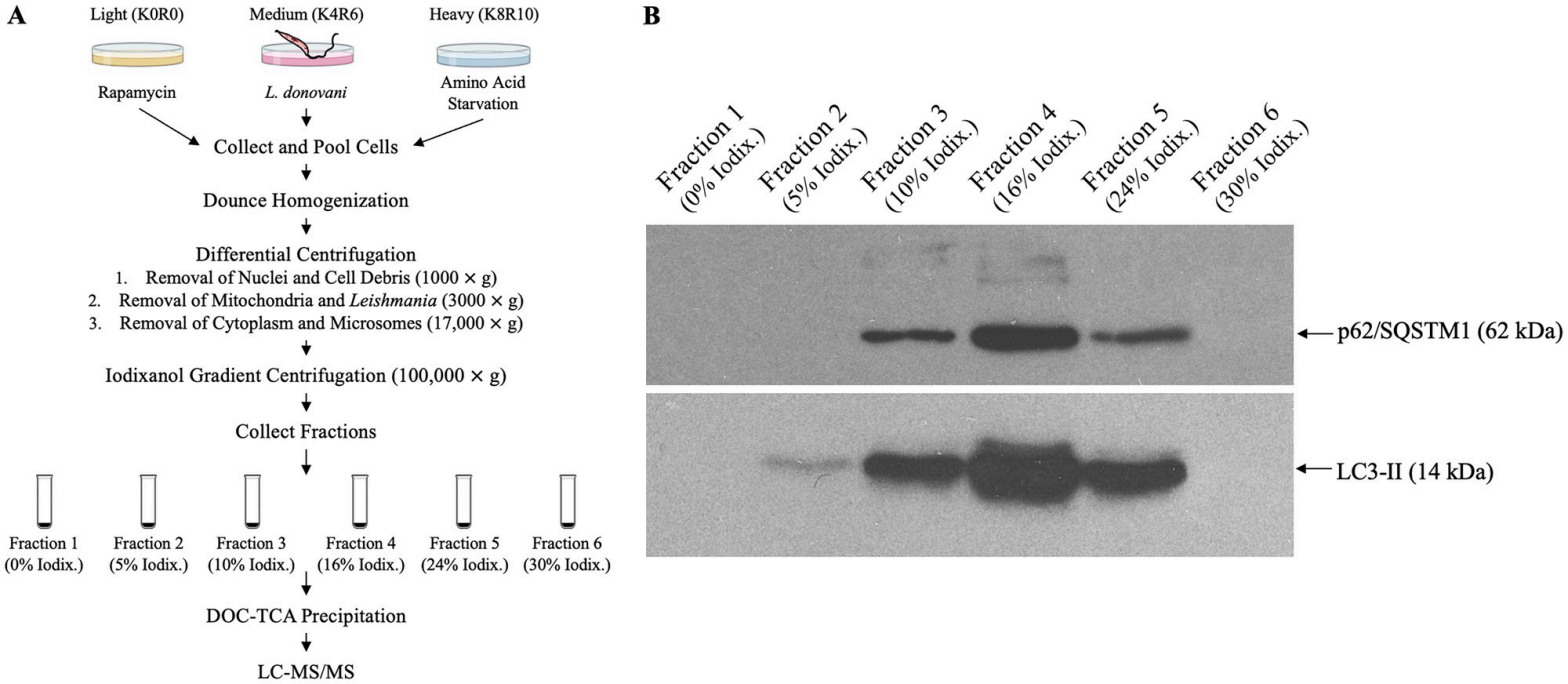
Preparation of samples for LC/MS analysis. A) Flow diagram outlining the autophagosome purification workflow in preparation for mass spectrometry analysis. THP-1 cells were stable isotope-labeled, differentiated, and subsequently treated with either 12.5 μg/mL rapamycin for 2 h, infected with *Leishmania donovani* at an MOI of 20:1 for 24 h or starved with HBSS for 4 h and treated with 100 nM Bafilomycin A1 for the last 3 h. To minimize quantitative error, the cells were mixed at an early stage and lysed via Dounce homogenization. Autophagosomes were purified via differential centrifugation, followed by iodixanol gradient centrifugation. Six fractions were collected, and samples were concentrated by 10% DOC-TCA precipitation. Fraction four (16% iodixanol) was subjected to LC/MS analysis. B) Western blot analysis of 10% DOC-TCA precipitated iodixanol gradient fractions for the autophagosome marker LC3-II and SQSTM1/p62. A representative image from three independent experiments is shown.

Despite the extensive progress that has been made in the field of autophagy, no study reported thus far has characterized the proteome of intracellular pathogen-induced autophagosomes [28, 37–40]. As a result, this study was designed to capture the global proteome of autophagosomes to identify proteins that are potentially modulated in *Leishmania*-induced autophagosomes. Here, we characterized both autophagosome membrane proteins and cargo proteins to gain insight into cellular proteome dynamics during the process of autophagy. To examine the possible selectivity of *Leishmania*-induced autophagosomes, its protein constituents were compared with those of autophagosomes induced by either rapamycin (an inhibitor of mTORC1) [41] or amino acid starvation [42, 43] in dTHP-1 cells. For this comparison, we used quantitative MS-based proteomics in conjunction with protein correlation profiling (PCP) [44] and SILAC [40, 45]. As outlined in Fig 1A and described in the materials and methods, THP-1 cells were grown in three separate SILAC media formulations: “light”, “medium”, and “heavy”. Cells were separately differentiated and autophagy was subsequently induced by rapamycin (selective autophagy) [41], *Leishmania* infection, or amino acid starvation (non-selective autophagy) [42, 43]. Autophagosome accumulation in the starvation condition was increased by treatment with Bafilomycin A1, an autophagy inhibitor that prevents the maturation of autophagic vacuoles. Following treatments, cells were processed for the isolation of autophagosomes using iodixanol gradient centrifugation and a total of six fractions (0%, 10%, 16%, 24%, and 30% iodixanol) were collected. Induction of autophagy in each treatment group was confirmed by Western blotting for LC3-II (S2 Fig). Furthermore, to rule out the possibility that *Leishmania*-induced autophagy is due to the general phagocytosis of external particles, we incubated dTHP-1 cells with latex beads at different MOIs for 24 h. Western blot analysis of the bead-treated cells for LC3-II levels revealed no significant induction of autophagy compared to non-treated control cells (S3 Fig). This strongly suggests that the induction of autophagy following *Leishmania* infection is not mediated by general phagocytotic processes.

To validate the autophagosome isolation procedure, the resulting fractions were analyzed via Western blot for the enrichment of the established autophagosome markers LC3-II and p62/SQSTM1 (Fig 1B). This analysis revealed that autophagosomes were mainly distributed in fractions three to five, with a majority residing in fraction four (Fig 1B). As a result, fraction four (autophagosome-enriched fraction) was subsequently used for mass spectrometry analysis. The proteome of autophagosomes from *Leishmania*-infected dTHP-1 cells was identified by filtering with Perseus (v1.6.15.0) as described in materials and methods. After filtering, 1787 proteins were identified as constituents of *Leishmania*-infected autophagosomes (S2 Table).

### Overview of identified proteins from *Leishmania*-induced autophagosomes

**Fig 2:**
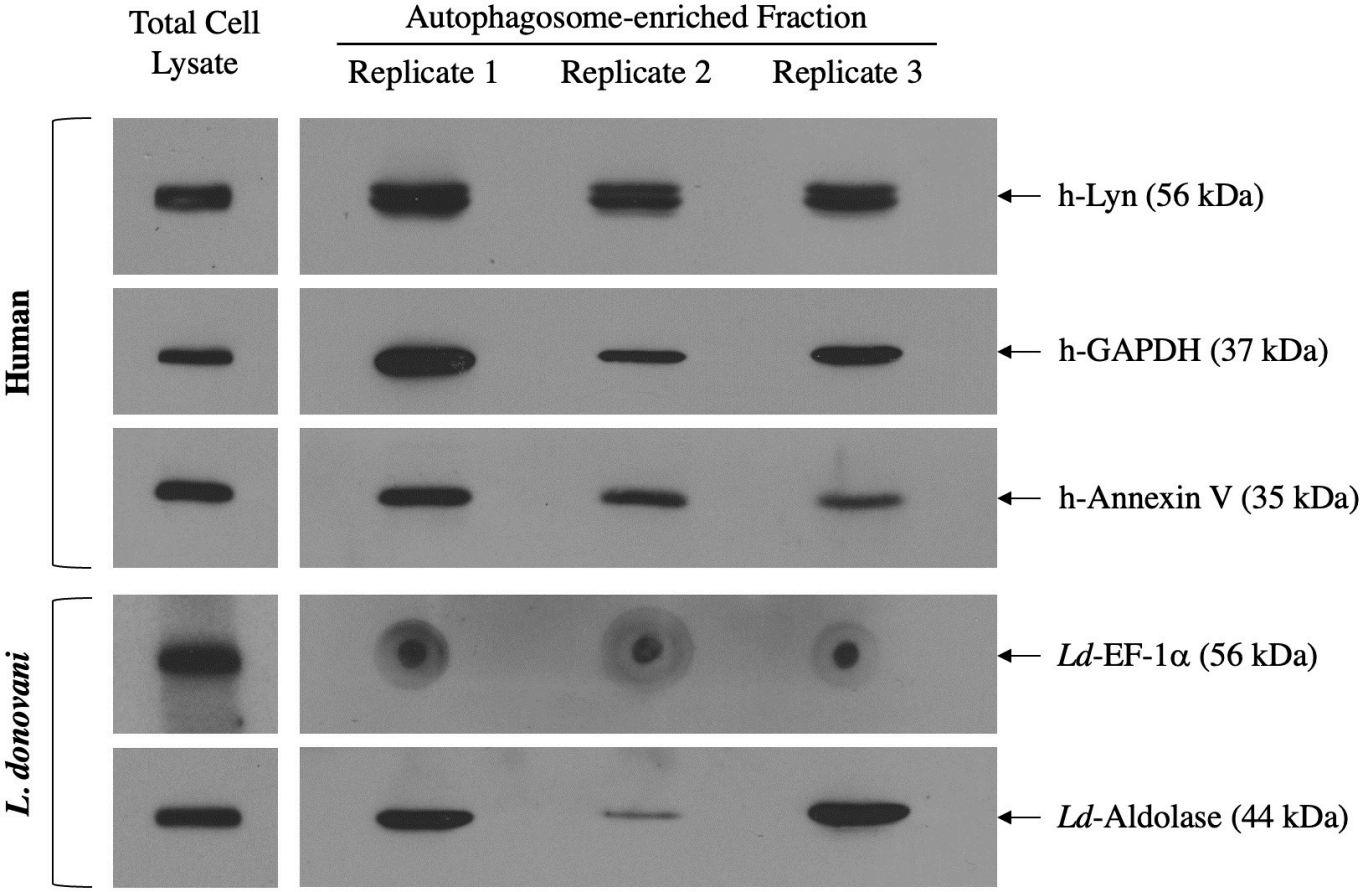
Validation of proteins identified by mass spectrometry analysis. Western blots of human Lyn, Annexin V, GAPDH, and *Leishmania* fructose bisphosphate aldolase (*Ld*-aldolase) from autophagosome-enriched fraction four as well as a dot blot of *Leishmania* EF-1α (*Ld*-EF-1α) from autophagosome-enriched fraction four (n = 3). Western blots for the respective proteins from total cell lysates were used as a positive control.

To independently validate the mass spectrometric data, we performed Western blot analyses of select proteins from the list of 1787 autophagosomal proteins from *Leishmania*-infected cells. The proteins were selected based on antibody availability and differences in molecular weight to ensure proper separation and identification via Western blot. Our data shows that the autophagosome-enriched fraction in all three replicates contained human GAPDH, Annexin V, and tyrosine-protein kinase Lyn (Fig 2), which is consistent with the mass spectrometry results.

**Fig 3:**
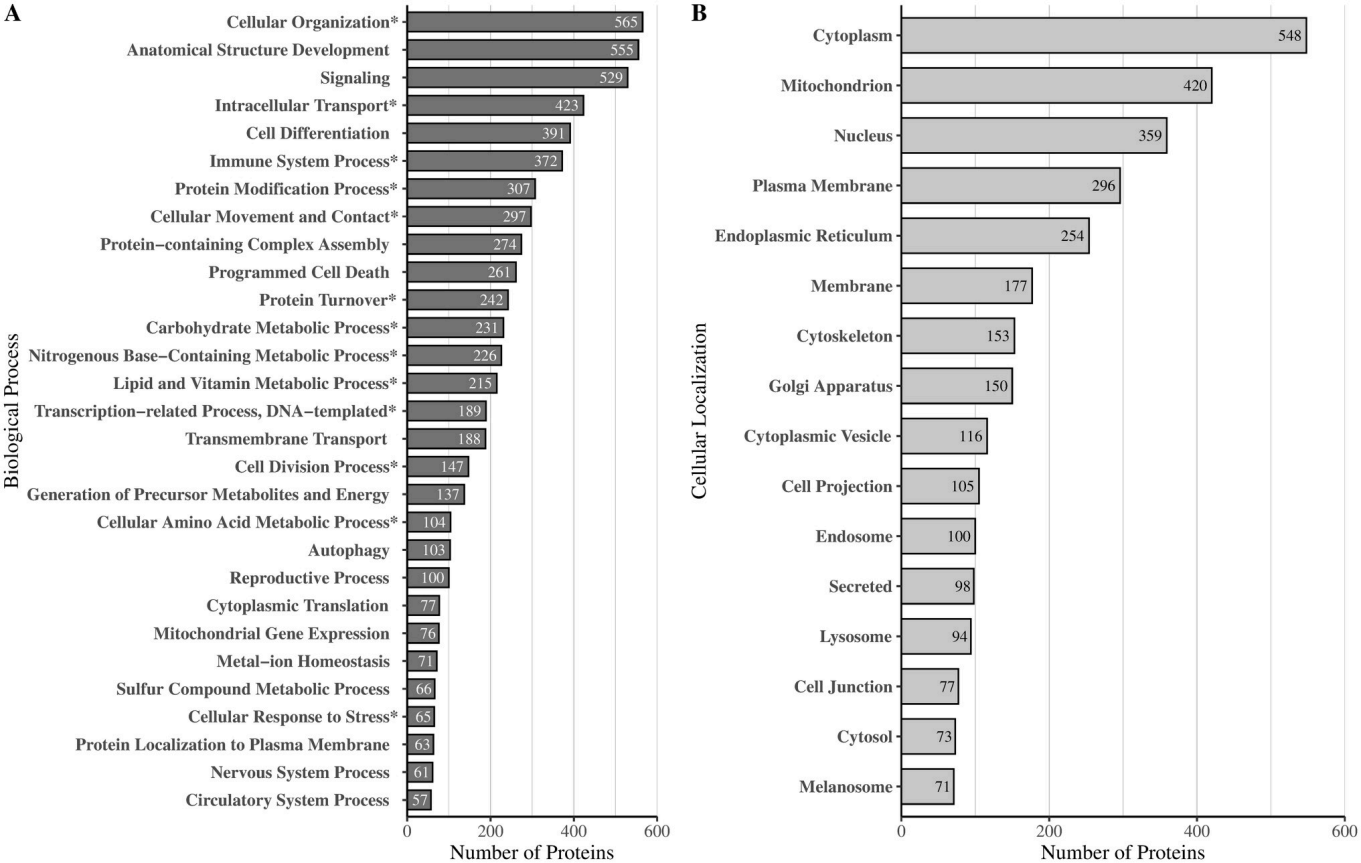
Gene ontology analysis of autophagosome-associated proteins from *Leishmania*-infected dTHP-1 cells. Proteins are categorized according to A) Biological Processes or B) Cellular Localization. The bars represent the number of proteins associated with each gene ontology (GO) term. Starred (*) gene ontology terms represent categories that encompass two or more related GO terms. Proteins with an organelle membrane or sub-organelle structure localization were relabeled as the corresponding organelle. The combined categories are outlined in S1 Table.

To determine how *Leishmania*-infected autophagosome-associated proteins may be involved in specific biological processes, we first classified the proteins into broad, process-based categories according to gene ontology (GO) annotations. To do this, we mapped the proteins to GO terms using the Generic Gene Ontology Term Mapper by Princeton University (https://go.princeton.edu/cgi-bin/GOTermMapper). For brevity, these terms were further grouped based on the similarity between biological processes (S1 Table). According to GO analysis, most proteins were associated with cellular development, anatomical structure development, and signaling (Fig 3A). The immune system process was also quite prevalent as were various metabolic processes such as carbohydrate, nitrogenous-base containing, lipid, and vitamin metabolic processes. Of note, we were able to identify 103 autophagy-related proteins (Fig 3A). This lends further support to the successful isolation of autophagosomes, despite the absence of LC3-II and ATG proteins from the mass spectrometry analysis. We also wanted to determine the subcellular origin of these autophagosome-associated proteins. We categorized the proteins based on their labeled subcellular locations obtained through the UniProtKB database and found that *Leishmania*-induced autophagosome-associated proteins were primarily localized to the cytoplasm, mitochondria, nucleus, and cell membrane (Fig 3B).

Interestingly, 23 leishmanial proteins were also identified as constituents of *Leishmania*-induced autophagosomes (Table 1). Strikingly, 13 of these proteins were previously identified as part of the *Leishmania* secretome [46]. Among them are *Ld*-EF-1α and *Ld*-aldolase, proteins which have been previously implicated in *Leishmania* pathogenesis [36, 47]. Due to the limited availability of *Leishmania*-specific antibodies against the list of detected *Leishmania* proteins, only *Ld*-aldolase and *Ld*-EF-1α were identified by Western blotting. Western blot analysis confirms the presence of *Ld*-aldolase in the autophagosome-enriched fraction, while the presence of *Ld*-EF-1α was confirmed using a dot blot assay (Fig 2).

**Table 1:** *Leishmania* proteins associated with host-cell autophagosomes.

Gene Name	Protein ID	Protein Name
*LDBPK_211170*	E9BFA3	Histone H2A
*LDBPK_170170*	A0A0R4J963	Elongation factor 1-alpha
*LDBPK_050510*	E9B8E1	ATPase alpha subunit
*LDBPK_051190*	E9B8K9	Prefoldin subunit, putative
*LDBPK_071340*	E9B9C1	Amino acid transporter, putative
*LDBPK_100960*	E9BAE1	Small GTP-binding protein Rab11, putative
*LDBPK_130330*	E9BBA8	Tubulin alpha chain
*LDBPK_190200*	E9BE16	ADP/ATP translocase
*LDBPK_190710*	E9BE66	Glycosomal Malate dehydrogenase
*LDBPK_201350*	E9BET4	Calpain-like cysteine peptidase, putative
*LDBPK_210300*	E9BF15	Hexokinase
*LDBPK_240140*	E9BGM6	Glycosomal membrane like protein
*LDBPK_251210*	E9BHM5	ATP synthase subunit beta
*LDBPK_261220*	E9BID4	Heat shock protein 70-related protein
*LDBPK_072500*	E9BJI0	Phosphoenolpyruvate carboxykinase (ATP)
*LDBPK_282430*	E9BJW3	Glycosomal membrane protein, putative
*LDBPK_302990*	E9BM42	Glyceraldehyde-3-phosphate dehydrogenase
*LDBPK_320520*	E9BNF6	Ras-related rab-4, putative
*LDBPK_331940*	E9BQC4	Small GTP-binding protein Rab18, putative
*LDBPK_340140*	E9BQG8	Malate dehydrogenase
*LDBPK_350150*	E9BRP7	Uncharacterized protein
*LDBPK_353750*	E9BSP6	Gim5A protein, putative
*LDBPK_361320*	E9BTJ1	Fructose-bisphosphate aldolase

### Stimuli-dependent modulation of autophagosome-associated proteome

Autophagy can be induced by a variety of stress-related stimuli that involve various mTOR-dependent and -independent signaling pathways. As we have previously shown, *Leishmania* induces mTOR-independent autophagy [4]. Hence, it was of interest to evaluate whether *Leishmania* alters the composition and abundance of autophagosome-associated proteins to satisfy the parasite’s nutritional needs for survival within the hostile environment of host cell phagolysosomes. For this evaluation, we compared protein abundances directly between autophagosomes isolated from *Leishmania*-infected, starved, and rapamycin-treated dTHP-1 cells. Interestingly, our PCP-SILAC-based analysis revealed stimuli-dependent modulations in select protein groups within the autophagosome-enriched fraction. After filtering through the MaxQuant (v1.5.1.0) generated protein ratios using Perseus, 1281 and 1275 proteins were retained in the lists of *Leishmania*- to rapamycin-induced and *Leishmania*- to starvation-induced ratios, respectively.

**Fig 4:**
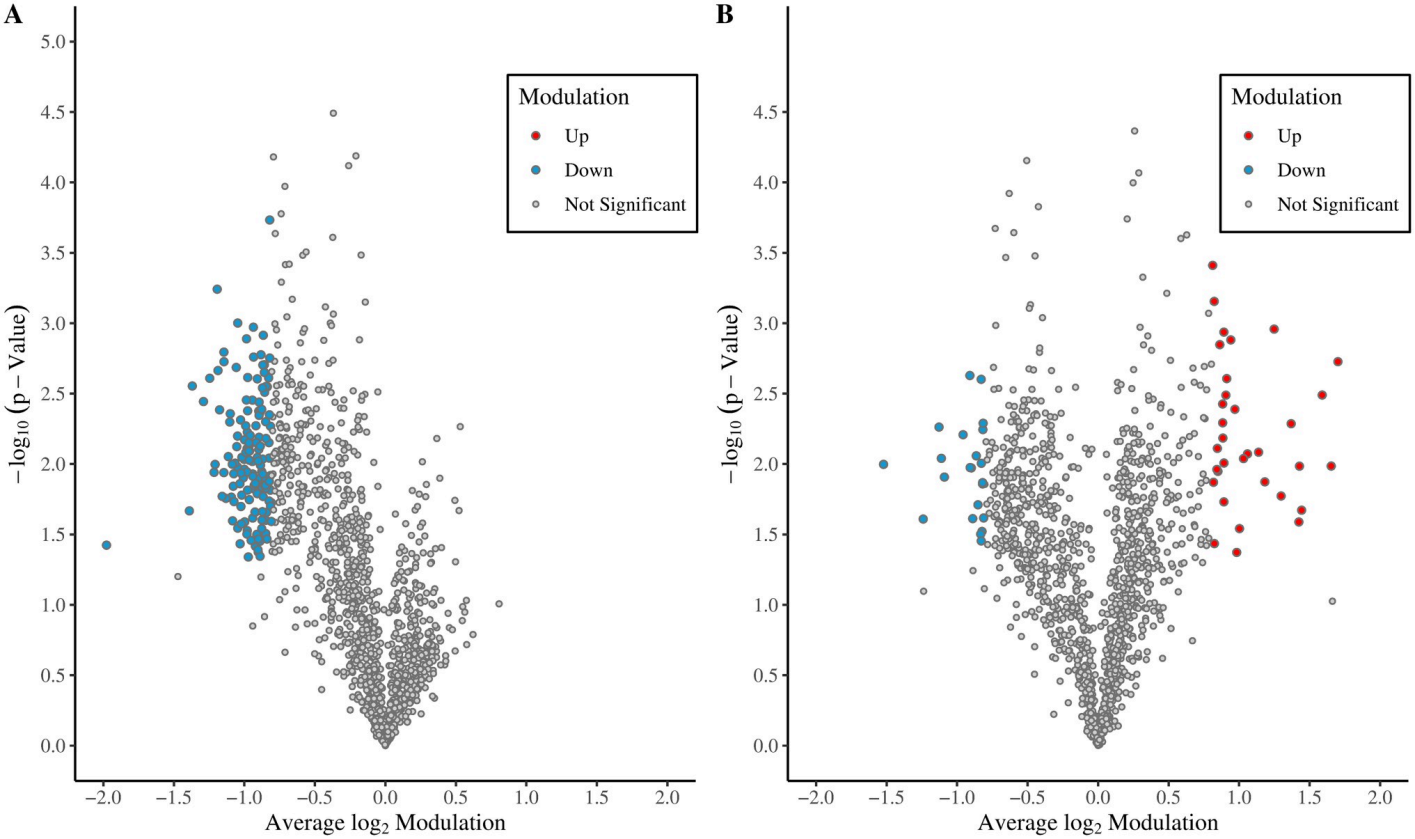
Volcano plot obtained from 3-plex SILAC-based quantitative analysis of autophagosome-associated proteins. The average of three log_2_ transformed *Leishmania*-infected/ rapamycin-treated (A) or *Leishmania*-infected/starvation SILAC protein ratios (B). Red and blue dots represent proteins exhibiting significant fold changes (at least 75%) up and down respectively, p < 0.05, n = 3.

Next, we examined whether any of these identified proteins were significantly modulated. Only proteins modulated by at least 75% and to a statistically significant degree (p < 0.05) were considered to be modulated. The data was visualized as a volcano plot to highlight the overall trend in protein modulations between each condition (Fig 4). Between *Leishmania*- and rapamycin-induced autophagy, 146 autophagosome-associated proteins were significantly modulated (Table 2, S3 Table). As shown by the volcano plot (Fig 4A), there appears to be a sizable subset of proteins that have quantitatively decreased. In contrast, when comparing *Leishmania*- and starvation-induced autophagosome-associated proteins, quantitative protein modulations occurred in both directions (Fig 4B). Of the 57 proteins which were modulated, 34 proteins were up-modulated while 23 were down-modulated (Table 3, Fig 4B).

**Table 2:** Twenty most modulated autophagosome-associated proteins in *Leishmania*-infected compared to rapamycin-treated dTHP-1.

Gene names	Protein ID	Protein names	Average Log2 Modulation
*ALCAM*	Q13740	CD166 antigen	-1.97819
*NT5DC2*	Q9H857	5-nucleotidase domain-containing protein 2	-1.39046
*TFAM*	Q00059	Transcription factor A, mitochondrial	-1.36946
*MT-CO2*	P00403	Cytochrome c oxidase subunit 2	-1.29038
*TXNRD2*	Q9NNW7	Thioredoxin reductase 2, mitochondrial	-1.24594
*COX5B*	P10606	Cytochrome c oxidase subunit 5B, mitochondrial	-1.21505
*NDUFB9*	Q9Y6M9	NADH dehydrogenase [ubiquinone] 1 beta subcomplex subunit 9	-1.20789
*MMAB*	Q96EY8	Cob(I)yrinic acid a,c-diamide adenosyltransferase, mitochondrial	-1.19304
*UQCRFS1; UQCRFS1P1*	P47985	Cytochrome b-c1 complex subunit Rieske, mitochondrial; Cytochrome b-c1 complex subunit 11; Putative cytochrome b-c1 complex subunit Rieske-like protein 1	-1.18724
*HSPE1; HSPE1-MOB4*	P61604	10 kDa heat shock protein, mitochondrial	-1.17613
*GRPEL1*	Q9HAV7	GrpE protein homolog 1, mitochondrial	-1.15765
*MRPL18*	Q9H0U6	39S ribosomal protein L18, mitochondrial	-1.14571
*MRPL37*	Q9BZE1	39S ribosomal protein L37, mitochondrial	-1.14537
*HSPD1*	P10809	60 kDa heat shock protein, mitochondrial	-1.14483
*COX6C*	P09669	Cytochrome c oxidase subunit 6C	-1.13098
*C21orf33*	P0DPI2		-1.11397
*MRPL15*	Q9P015	39S ribosomal protein L15, mitochondrial	-1.10384
*ALDH18A1*	P54886	Delta-1-pyrroline-5-carboxylate synthase; Glutamate 5-kinase; Gamma-glutamyl phosphate reductase	-1.10036
*MRPS7*	Q9Y2R9	28S ribosomal protein S7, mitochondrial	-1.09256
*MRPL11*	Q9Y3B7	39S ribosomal protein L11, mitochondrial	-1.08601

**Table 3:** Twenty most modulated autophagosome-associated proteins in *Leishmania*-infected compared to starved dTHP-1.

Gene names	Protein ID	Protein names	Average Log2 Modulation
*PPT1*	P50897	Palmitoyl-protein thioesterase 1	2.072375
*ITGAM*	P11215	Integrin alpha-M	1.701131
*F11R*	Q9Y624	Junctional adhesion molecule A	1.653624
*ITGB7*	P26010	Integrin beta-7; Integrin beta	1.589531
*CTSB*	P07858	Cathepsin B; Cathepsin B light chain; Cathepsin B heavy chain	1.443597
*CD109*	Q6YHK3	CD109 antigen	1.428292
*TPP1*	O14773	Tripeptidyl-peptidase 1	1.424175
*CD9*	P21926	CD9 antigen; Tetraspanin	1.36924
*GLG1*	Q92896	Golgi apparatus protein 1	1.29827
*ANXA2; ANXA2P2*	P07355	Annexin A2; Annexin; Putative annexin A2-like protein	1.24884
*CTSD*	P07339	Cathepsin D; Cathepsin D light chain; Cathepsin D heavy chain	1.182757
*S100A10*	P60903	Protein S100-A10	1.138326
*LIPA*	P38571	Lysosomal acid lipase/cholesteryl ester hydrolase	1.059779
*ASPH*	Q12797	Aspartyl/asparaginyl beta-hydroxylase	1.031428
*ALDH2*	P05091	Aldehyde dehydrogenase, mitochondrial	-1.09031
*SOD2*	P04179	Superoxide dismutase [Mn], mitochondrial	-1.11184
*STMN1*	P16949	Stathmin	-1.12842
*LAPTM5*	Q13571	Lysosomal-associated transmembrane protein 5	-1.24044
*NDUFA4*	O00483	Cytochrome c oxidase subunit NDUFA4	-1.52182
*SQSTM1*	Q13501	Sequestosome-1	-2.43548

**Fig 5:**
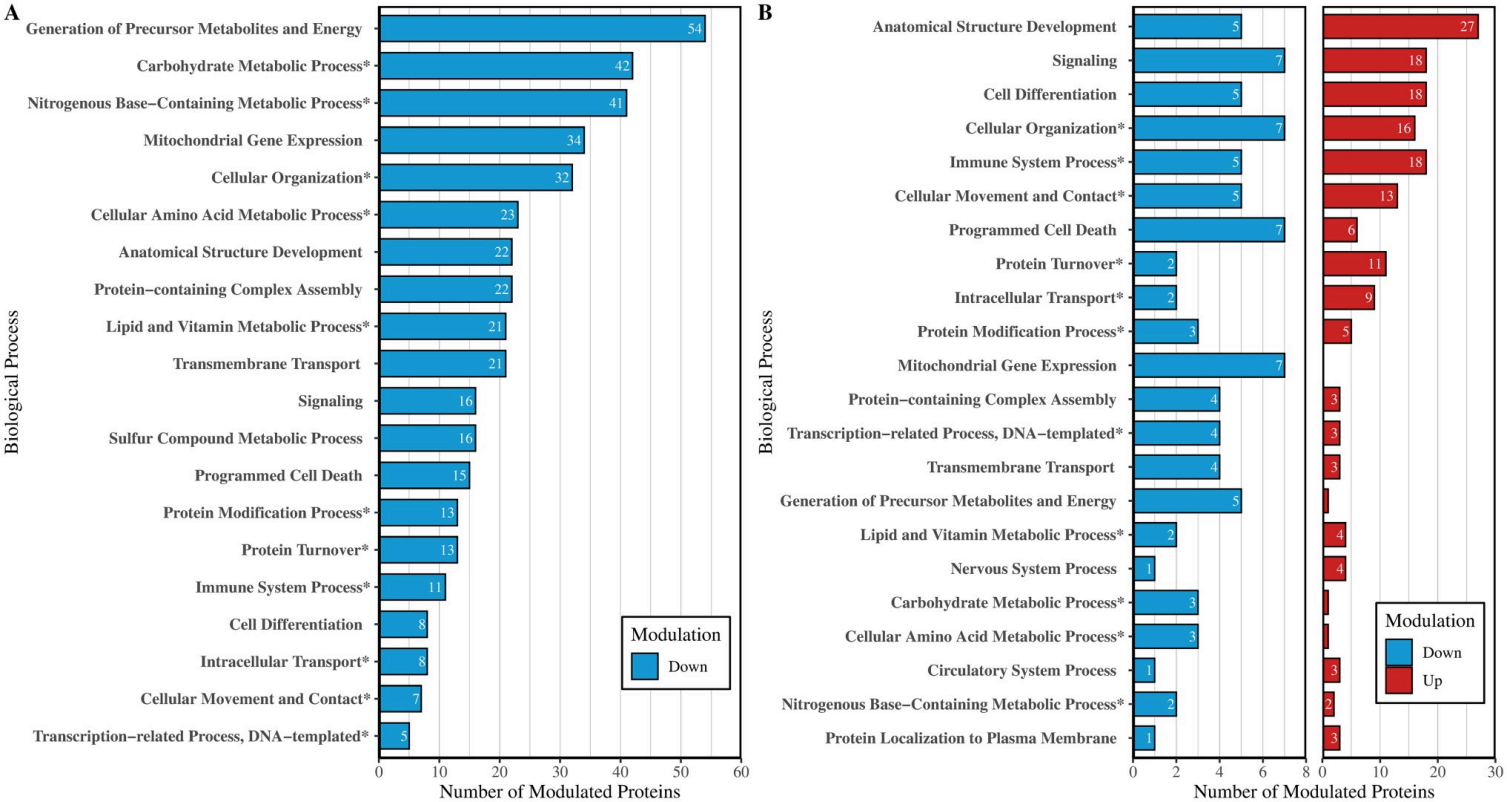
Gene ontology analysis of significantly modulated autophagosome-associated proteins. The Biological Processes associated with down-modulated (blue) and up-modulated (red) proteins from *Leishmania*-infected compared to rapamycin-treated conditions (A) or starvation conditions (B) are summarized above. The bars represent the number of proteins belonging to each GO term. Starred (*) gene ontology terms represent categories that encompass two or more related GO terms. The combined categories are outlined in S1 Table.

To gain insight into how these selectively modulated autophagosome-associated proteins may be involved in specialized cellular processes, we mapped the subset of significantly modulated proteins to GO terms using the GOTermMapper. Between the *Leishmania* infection and rapamycin-treatment conditions, most of the modulated proteins were associated with metabolic processes. 42 proteins were associated with carbohydrate metabolic process, 23 with cellular amino acid metabolic process, 41 with nitrogenous base-containing metabolic process, and 21 with lipid and vitamin metabolic processes (Fig 5A). This was expected since both rapamycin and *Leishmania* can alter the mTOR signaling pathway, which is involved in cell growth and metabolism [48]. Between the *Leishmania* infection and starvation treatment conditions, most of the modulated proteins were associated with anatomical structure development (Fig 5B), which was unexpected. However, many of these proteins were also associated with other GO terms such as signaling, cell differentiation, and immune system process.

## Discussion

Quantitative proteomic MS techniques like SILAC-MS/MS provide excellent sensitivity for protein detection and offer the capability for high-throughput, whole proteome analysis. It also allows samples to be mixed at early stages to minimize variations due to technical error and permits the comparison of multiple investigational conditions in a single MS run. Given these advantages, this method has the potential to uncover novel global and integrated views of biological mechanisms involved in pathogen biology and host-cell interactions [49–51]. However, very limited proteomic studies have been reported regarding the mechanisms involved in *Leishmania*-host cell interactions, and thus need to be comprehensively investigated.

Autophagy has been shown to play a role in intracellular pathogenesis [16]. In this regard, we have recently shown that *Leishmania* modulates host cell autophagy to facilitate parasite survival and proliferation [4]. In order to understand the mechanisms involved, our present study employed PCP-SILAC-based quantitative proteomic analysis to identify and characterize the proteome of *Leishmania*-induced host cell autophagosomes, including cargo and core membrane proteins (S2 Table). We identified 1787 proteins belonging to *Leishmania*-induced host cell autophagosomes. Analysis of the subcellular distribution of these proteins, revealed that many had a cytoplasmic localization which was expected for autophagosome-derived proteins. Many proteins also had a mitochondrial localization, which suggests the occurrence of autophagy-mediated mitochondrial turnover (mitophagy) (Fig 3B). We would like to note, however, that we cannot rule out that a portion of these mitochondrial proteins may have possibly originated from mitochondrial contamination during the isolation procedure. With our current methods, it will be technically very challenging to distinguish between proteins originating from mitophagic processes and those from contaminating mitochondria since the resulting proteome will likely be similar.

Interestingly, we also identified 23 *L*. *donovani* proteins within the autophagosome-enriched fraction. We considered the possibility that these *Leishmania* proteins may have originated from host-mediated *Leishmania* degradation. To investigate this, parasite burden assays were conducted at early and late time-points of infection. As shown in S1 Fig, we did not find a significant change in parasite burden from 3 to 24 hrs of infection, which suggests a negligible level of parasite clearance. Additionally, according to our previously published parasite rescue assays, a majority of parasites remain viable 24 hours post infection [4]. To supplement, the relatively short list of *Leishmania* proteins identified in this study, also argues against the occurrence of generalized parasite degradation. Although we cannot definitively rule out the possibility that a portion of *Leishmania* proteins may have originated from host-mediated intracellular parasite clearance, our data taken together suggests that this is unlikely a major contributor to the Leishmanial proteins detected in the autophagosomes [4]. Strikingly, over half of the Leishmanial proteins identified have been previously reported as part of the glycosome or secretome of *L*. *donovani* [46]. Among the identified *Leishmania* proteins are elongation factor-1 alpha and fructose bisphosphate aldolase, both of which have been implicated in *Leishmania* pathogenesis [36, 47]. In addition, we have also identified five *Leishmania* integral membrane proteins according to their UniProt annotations: amino acid transporter (*LDBPK_071340*), ADP/ATP translocase (*LDBPK_190200*), glycosomal membrane like protein (*LDBPK_240140*), glycosomal membrane protein (*LDBPK_282430*), and uncharacterized protein (*LDBPK_350150*). These membrane proteins may be able to integrate within autophagosome membranes to act as potential receptors.

Validation of proteomic data was done by SDS-PAGE western blot analysis on the autophagosome-enriched fraction for three human (GAPDH, Annexin V, and Lyn) and two *Leishmania* proteins (Fructose bisphosphate aldolase and EF-1α) (Fig 2). We note that *Leishmania* EF-1α was not detected by conventional Western blot analysis. The protein may be of low abundance within the sample and any additional protein loss accrued during electrophoresis and protein transfer may have further weakened the signal. Alternatively, EF-1α could have been degraded during sample preparation or storage. As a result, EF-1α protein fragments that our antibodies were produced against may have been lost during SDS-PAGE size-based protein separation. Instead, we used dot blot analysis for the detection of *Leishmania* EF-1α. The specificity of this antibody has been previously shown [36].

We then compared the protein profile of *Leishmania*-induced autophagosomes (24 hours) to the protein composition of autophagosomes induced by two well-characterized autophagy stimuli: amino acid starvation (2 hours) and rapamycin (4 hours). These autophagy treatment lengths were chosen to ensure the induction of an optimal autophagic response while avoiding excessive metabolic stress and cell damage. For example, we have previously shown that *Leishmania* optimally induces autophagy following 24 hours of infection [4]. However, rapamycin treatment and amino acid starvation for similar durations resulted in cell detachment (data not shown). Instead, shorter durations of treatment appear to be sufficient to induce clear autophagic responses and have been previously used in autophagy-related studies[4, 52–55]. However, we cannot rule out the possibility that these varying treatment lengths may have an impact on autophagosome composition. Nevertheless, the main focus of the study was to identify protein composition of autophagosomes induced by *Leishmania* infection.

Strikingly, when comparing the autophagosome proteomes of *Leishmania*-infected, rapamycin-treated and amino acid-starved cells, no unique proteins were found. The absence of unique proteins could be due to their low abundance in the sample and may require additional input material to enable detection by mass spectrometric analysis. Additionally, the signal of any unique membrane proteins may be masked by the abundance of cargo proteins. Many membrane proteins also tend to aggregate and precipitate in the solution, making sample preparation notoriously difficult.

Further statistical testing revealed 146 and 57 significantly modulated proteins in the *Leishmania*-to-rapamycin and *Leishmania*-to-starvation conditions, respectively. Since the number of modulated proteins was substantially lesser in the later comparison, this suggests that the kinetics of *Leishmania*-induced autophagy more closely resembles starvation-induced autophagy as opposed to selective, rapamycin-induced autophagy. This observation thus provides an interesting insight into how *Leishmania* modulates or induces autophagy. It is known that starvation-mediated autophagy can be induced through a mTORC1-dependent pathway [11, 42]. It also seems possible that the nutritional stress generated by *Leishmania* infection on host cells is not sufficient to negatively impact mTORC1 activity. Yet, it may be sufficient to induce mTORC1-independent, starvation-like autophagy required for delivering nutrients to *Leishmania* residing in the phagolysosome of host cells. The extensive similarities between *Leishmania*-induced autophagy and starvation-induced autophagy strongly suggest that *Leishmania*-induced mTOR-independent autophagy is not as selective as once thought. Together, it unveils a smart survival strategy adopted by *Leishmania* to promote autophagy while keeping the PI3K/Akt/mTORC1 pathway active, which is essential to inhibit host apoptotic induction. Indeed, it is known that *Leishmania* inhibits host macrophage apoptosis to promote its survival [56, 57].

Gene ontology analysis revealed that many of the significantly modulated autophagosome-associated proteins between *Leishmania*- and rapamycin-induced conditions were associated with metabolic processes (Fig 5A). This corroborates previous findings that show a link between rapamycin treatment and increased rates of mitochondrial autophagy (mitophagy) [58, 59]. Studies by Li et al. [58], and Wang et al. [59] found that in rapamycin-treated mice, there was an increase in the co-localization of LC3-II with the mitochondrial markers, VDAC-1(voltage-dependent anion-selective channel protein 1) or TOMM20 (translocase of outer mitochondrial membrane 20). Additionally, *Leishmania* infection has been shown to inhibit mTOR-dependent autophagy, which likely encompasses the process of mitophagy [4]. Together, both the activation of mitophagy by rapamycin treatment as well as the potential inhibition of mitophagy by *Leishmania* infection may have contributed to the large subset of down-modulated mitochondrial proteins observed in the dataset (Fig 4A). Conversely, gene ontology results show that many of the modulated autophagosome-associated proteins between *Leishmania*- and starvation-induced conditions were associated with several distinct biological processes (Fig 5B). Although anatomical function was the most prevalent GO term observed, many of those proteins were also associated with signaling, immune function, and cellular development. Because the proteome of *Leishmania*-induced autophagosomes appears to resemble starvation-induced autophagosomes, we proposed that *Leishmania*-induced autophagy, as in starvation conditions, may be non-selective. As a result, modulations in the proteome of autophagosomes may be reflective of modulations in the proteome of the cell at large. Indeed, *Leishmania*-infection has been shown to alter host-cell signaling and immune function [25, 60, 61] to promote its survival.

It was of interest to compare our dataset to previously published autophagosome compositions. A data set reported by Dengjel et al. [28] revealed the composition of autophagosomes induced by rapamycin, starvation, and concanamycin in MCF7-GFP cells. We compared our list of 1281 *Leishmania*-to-rapamycin proteins to their list of 359 rapamycin-induced autophagosome-associated proteins and found 152 proteins in common. Additionally, we compared our database with the 94 common autophagosomal proteins found across all inducers in the study by Dengjel et al. [28] and found 49 of those proteins in our proteome. Despite differences in cell type and equipment, the substantial degree of overlap validates our autophagosome isolation protocol. We note that the well-established autophagosomal marker protein, LC3-II [10, 62], was in insufficient quantity to be detected in our autophagosome-enriched samples but was clearly detectable by Western blotting (Fig 1B). We also compared our list of 1281 autophagosome-associated proteins to a dataset published by Øverbye et al. [39] that consisted of 39 autophagosome membrane-enriched proteins in rat hepatocytes and found 13 proteins in common. Interestingly, Øverbye et al. were also unable to detect LC3-II, although the presence of the protein was detected through Western blotting.

While our data provides an extensive list of autophagosome-associated proteins, it likely is not exhaustive. This protocol is unable to detect autophagosomal proteins that are not expressed in dTHP-1 cells, are inactive under our experimental conditions, are in insufficient quantity, or were detached from the autophagosomes during the isolation protocol. Nevertheless, the proteome of autophagosomes isolated from *Leishmania*-infected host cells is comprised of a complex mixture of proteins enriched in autophagosome-related activities, which highlights the intricate nature of this degradative pathway that we are only beginning to understand. We also note that our data set is restricted to dTHP-1 cells and *L*. *donovani* parasites. It will be of interest to validate our findings in primary human macrophages. However, as conventional SILAC protocols require the active proliferation of cells for the complete metabolic labeling of proteins, it will be challenging to perform SILAC-coupled LC-MS/MS in non-dividing cells like primary human macrophages. Instead, alternative methods may be considered for future studies.

In summary, this study presents the first global protein profile of autophagosomes during intracellular parasitic infection. PCP-SILAC-based quantitative analysis of autophagosomes isolated from THP-1 cells subjected to three different stimuli revealed that the composition of *Leishmania*-induced autophagosomes was more similar to starvation- rather than rapamycin-induced autophagosomes, which suggests the non-selective nature of *Leishmania* induced autophagy. Interestingly, we also identified *Leishmania* proteins within the autophagosome-enriched fraction from *Leishmania* infected hosts. Although the experimental design used for this study does not allow us to directly distinguish between cargo and core membrane proteins, the proteomic data presented above provide a framework of *Leishmania*-induced autophagosome-associated proteins that will help in characterizing the underlying cellular processes involved in the interaction of *Leishmania* with the host cell.

## Supporting information

S1 Fig Analysis of parasite burden in *L*. *donovani*-infected dTHP-1 cells. dTHP-1 cells were incubated with stationary phase *L*. *donovani* for 3 hrs and washed to remove uninternalized parasites. Infected cells were either immediately fixed with paraformaldehyde or incubated for an additional 21 hrs, for a total infection time of 24 hrs, before fixing. Cells were stained with DAPI and fluoresence microscopy images were taken using the Zeiss Axioplan 2 imaging microscope. Bars represent mean (+/- SE) parasite burden (parasite count per 100 macrophages) at 3 hrs and 24 hrs. Statistical analysis (t-test) revealed no signicant difference between the means.
(TIF)

S2 Fig Increase in LC3-II following stimulation with autophagy inducers. dTHP-1 cells were incubated with *L*. *donovani* promastigotes (MOI of 20:1) for 24 h, rapamycin (12.5μg/mL) for 2 h, or starved in HBSS media for 4 h with and without bafilomycin A1 (100nM) for the final 3 h. Whole cell lysates of the control and treated cells were collected and analyzed using Western blot for LC3-II. Actin was used as a loading control.
(TIF)

S3 Fig Increase in LC3-II following *Leishmania* infection is not mediated by phagocytosis. dTHP-1 cells were incubated with latex beads at a bead-to-cell ratio of 5:1, 10:1 and 20:1 for 24 h to elicit phagocytosis and bead internalization. Treatment of cells with 12.5 μg/mL of rapamycin for 2h serves as a positive control. Whole cell lysates were collected and analyzed using Western blot for LC3-II. Actin was used as a loading control. A representative image of three independent experiments is shown.
(TIF)

S1 Table Combined gene ontology categories. (XLSX)

S2 Table *Leishmania*-induced autophagosome-associated proteins (M). (XLSX)

S3 Table Significantly modulated autophagosome-associated proteins in *Leishmania*-infected (M) compared to rapamycin-treated (L) dTHP-1. (XLSX)

S4 Table Significantly modulated autophagosome-associated proteins in *Leishmania*-infected (M) compared to starved (H) dTHP-1. (XLSX)

S1 Raw images Raw western blot images. (PDF)
