# Supplementary figures and images for: Comprehensive proteomic analysis of autophagosomes derived from Leishmania-infected macrophages
Source: PLoS One. 2023 Apr 7;18(4):e0284026. doi: 10.1371/journal.pone.0284026 (PMC10081754; doi:10.1371/journal.pone.0284026)

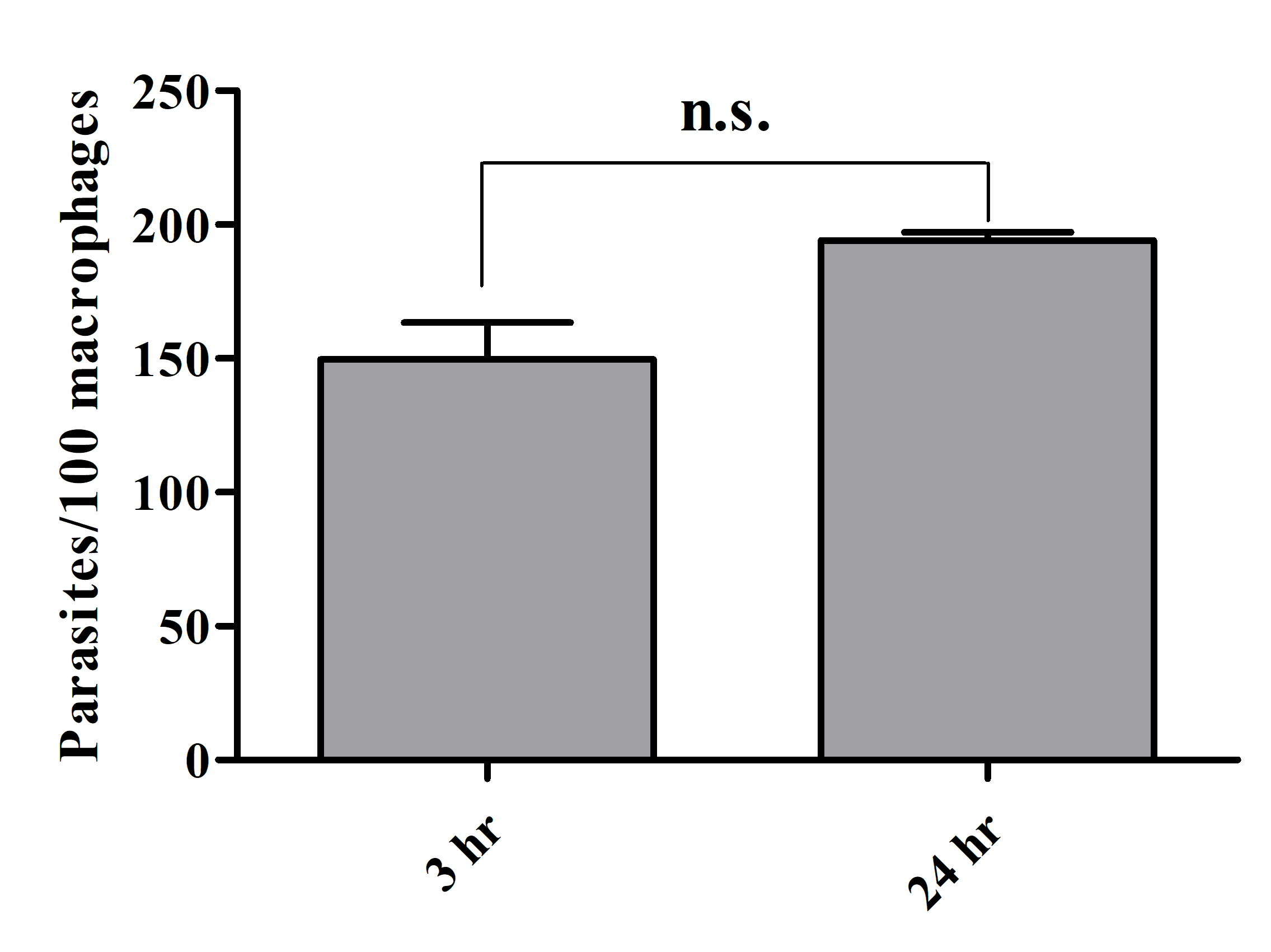

Supplement: S1 Fig — dTHP-1 cells were incubated with stationary phase L. donovani for 3 hrs and washed to remove uninternalized parasites. Infected cells were either immediately fixed with paraformaldehyde or incubated for an additional 21 hrs, for a total infection time of 24 hrs, before fixing. Cells were stained with DAPI and fluoresence microscopy images were taken using the Zeiss Axioplan 2 imaging microscope. Bars represent mean (+/- SE) parasite burden (parasite count per 100 macrophages) at 3 hrs and 24 hrs. Statistical analysis (t-test) revealed no signicant difference between the means. (TIF) [file pone.0284026.s001.tif]

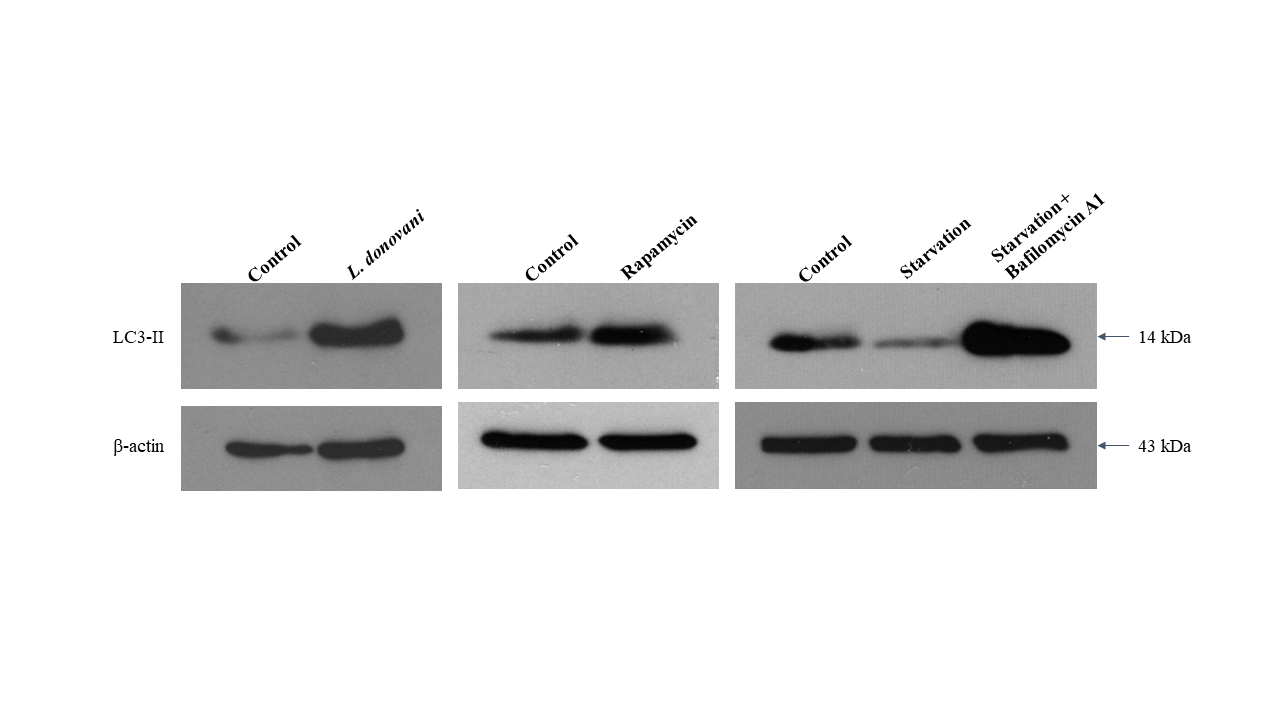

Supplement: S2 Fig — dTHP-1 cells were incubated with L. donovani promastigotes (MOI of 20:1) for 24 h, rapamycin (12.5μg/mL) for 2 h, or starved in HBSS media for 4 h with and without bafilomycin A1 (100nM) for the final 3 h. Whole cell lysates of the control and treated cells were collected and analyzed using Western blot for LC3-II. Actin was used as a loading control. (TIF) [file pone.0284026.s002.tif]

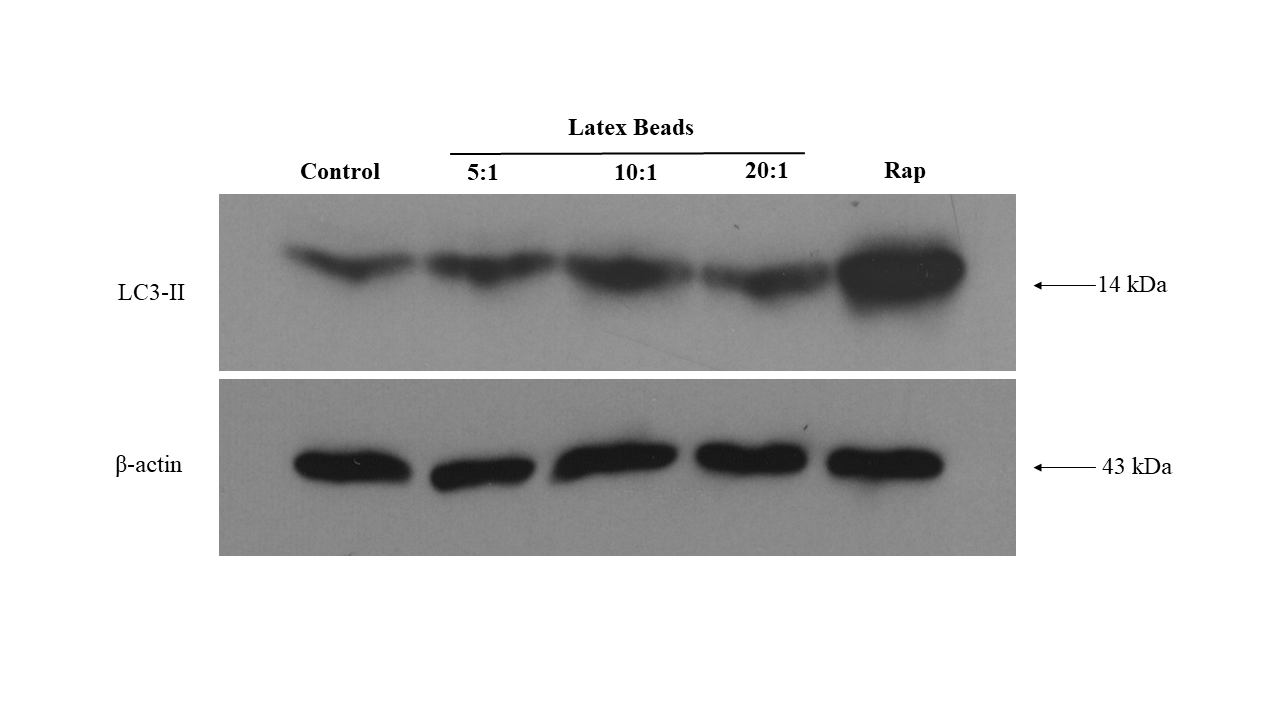

Supplement: S3 Fig — dTHP-1 cells were incubated with latex beads at a bead-to-cell ratio of 5:1, 10:1 and 20:1 for 24 h to elicit phagocytosis and bead internalization. Treatment of cells with 12.5 μg/mL of rapamycin for 2h serves as a positive control. Whole cell lysates were collected and analyzed using Western blot for LC3-II. Actin was used as a loading control. A representative image of three independent experiments is shown. (TIF) [file pone.0284026.s003.tif]
