# Supplementary material for: Comprehensive proteomic analysis of autophagosomes derived from Leishmania-infected macrophages
Source: PLoS One. 2023 Apr 7;18(4):e0284026. doi: 10.1371/journal.pone.0284026 (PMC10081754; doi:10.1371/journal.pone.0284026)
Supplement: S1 Raw images — (PDF) [file pone.0284026.s008.pdf]

Fig 1B. LC3-II Western Blot of all fractions from iodixanol density gradient

15% Tris-Tricine SDS-PAGE
PVDF

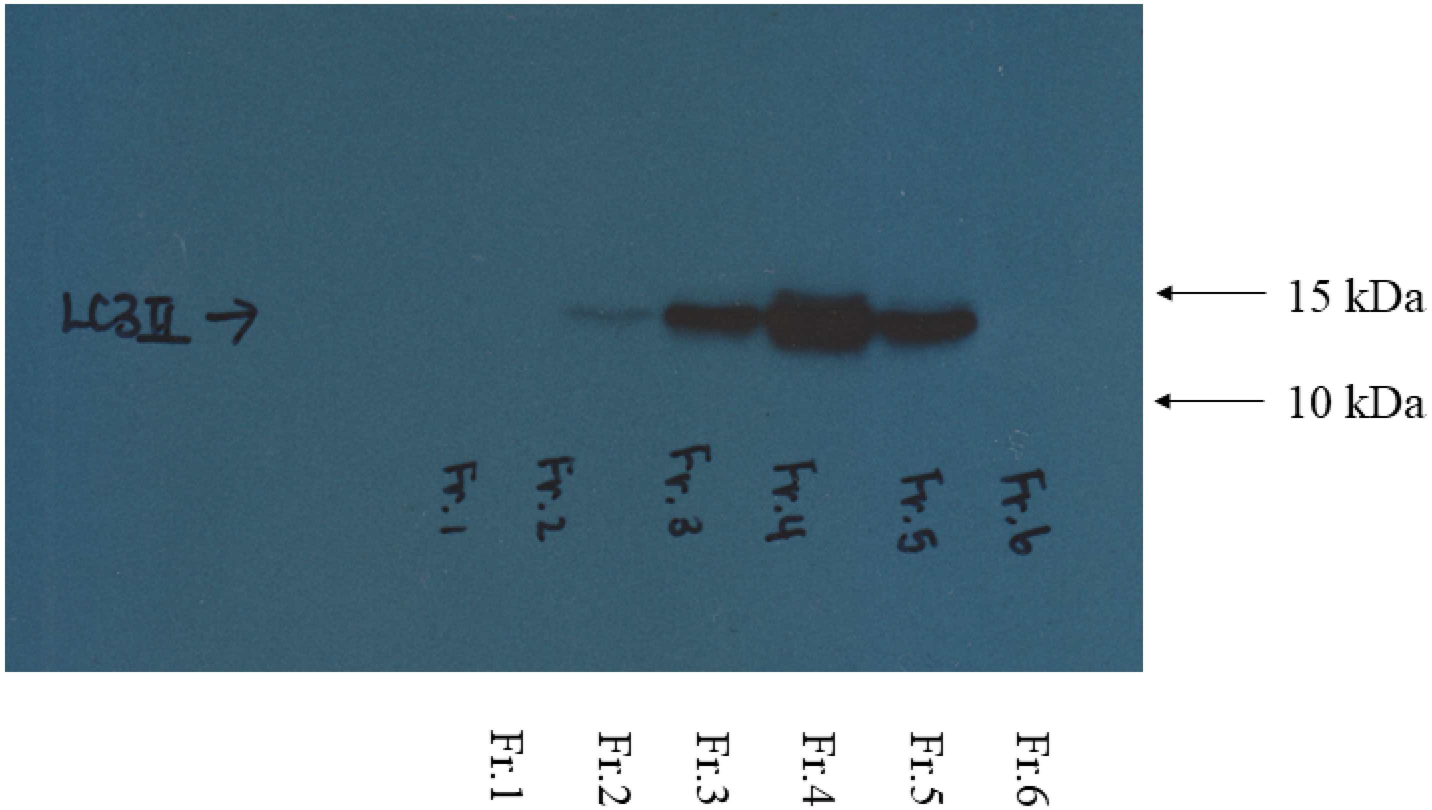

Image captured via ECL and X-ray film

Fig 1B. p62/SQSTM1 Western Blot of all fractions from iodixanol density gradient

15% Tris-Tricine SDS-PAGE
PVDF

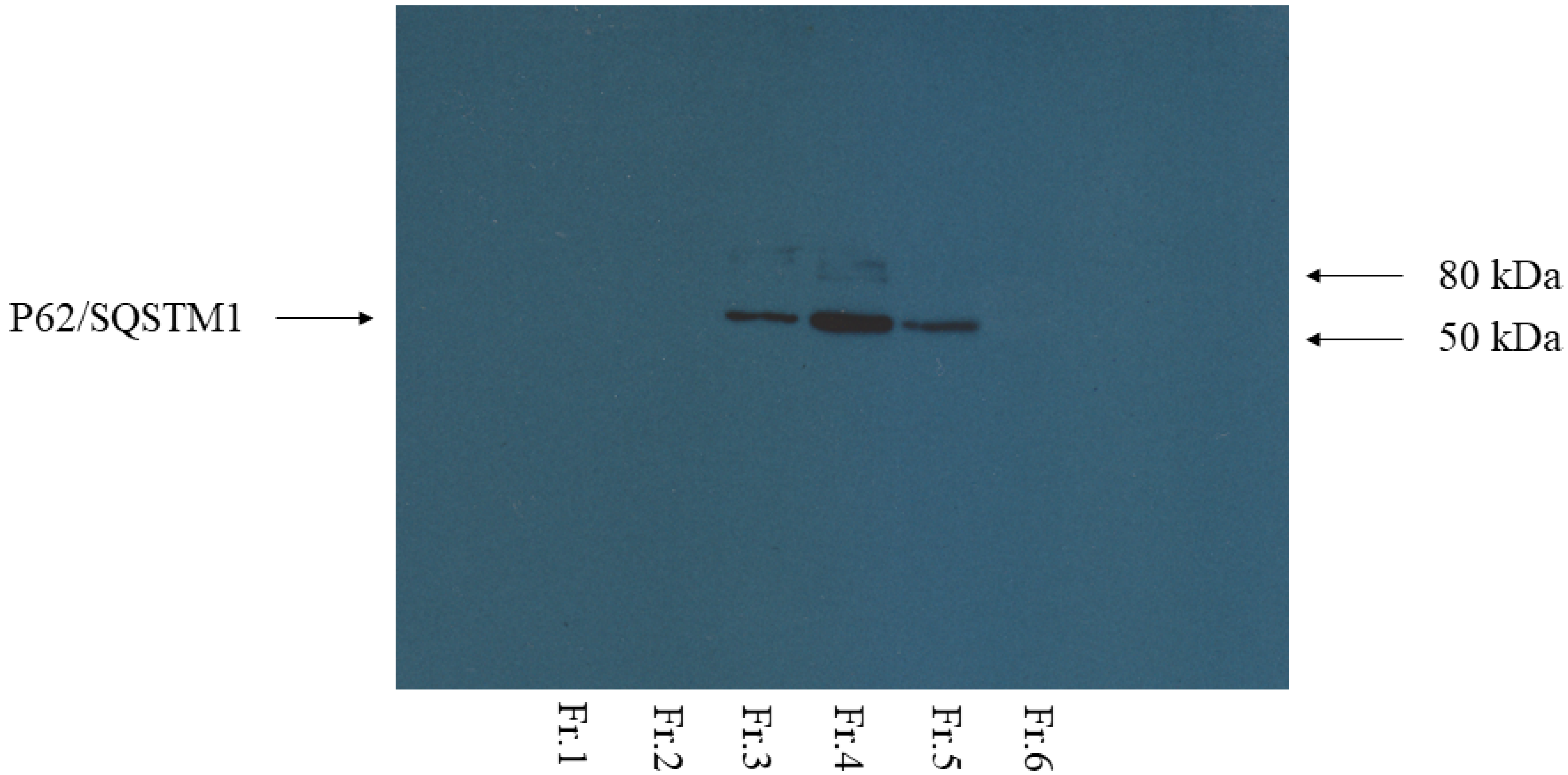

Image captured via ECL and X-ray film

Fig 2. LYN Western Blot of autophagosome-enriched fractions from all three replicates

10% Tris-Glycine SDS-PAGE
Nitrocellulose

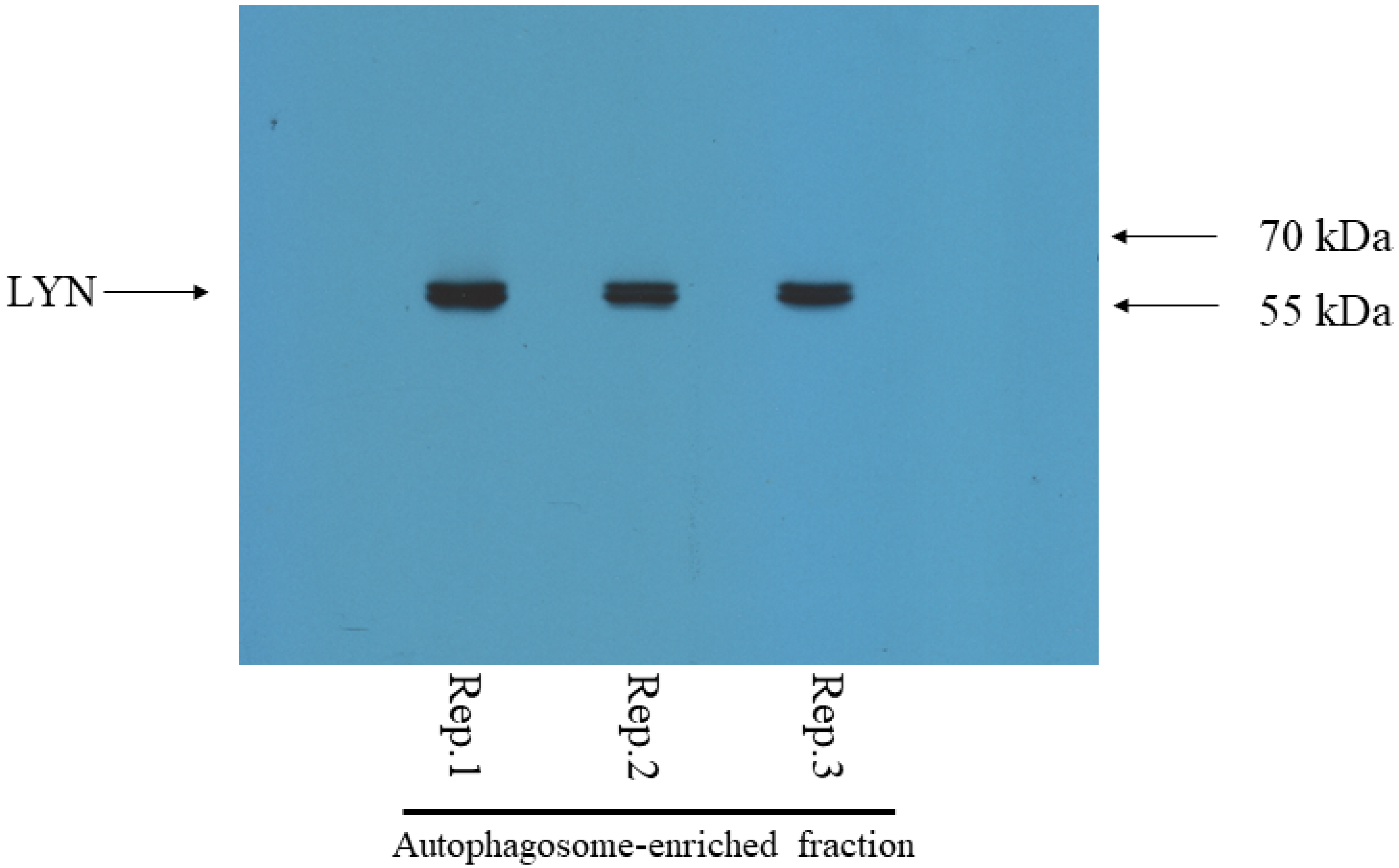

Image captured via ECL and X-ray film

Fig 2. GAPDH Western Blot of autophagosome-enriched fractions from all three replicates

10% Tris-Glycine SDS-PAGE
Nitrocellulose

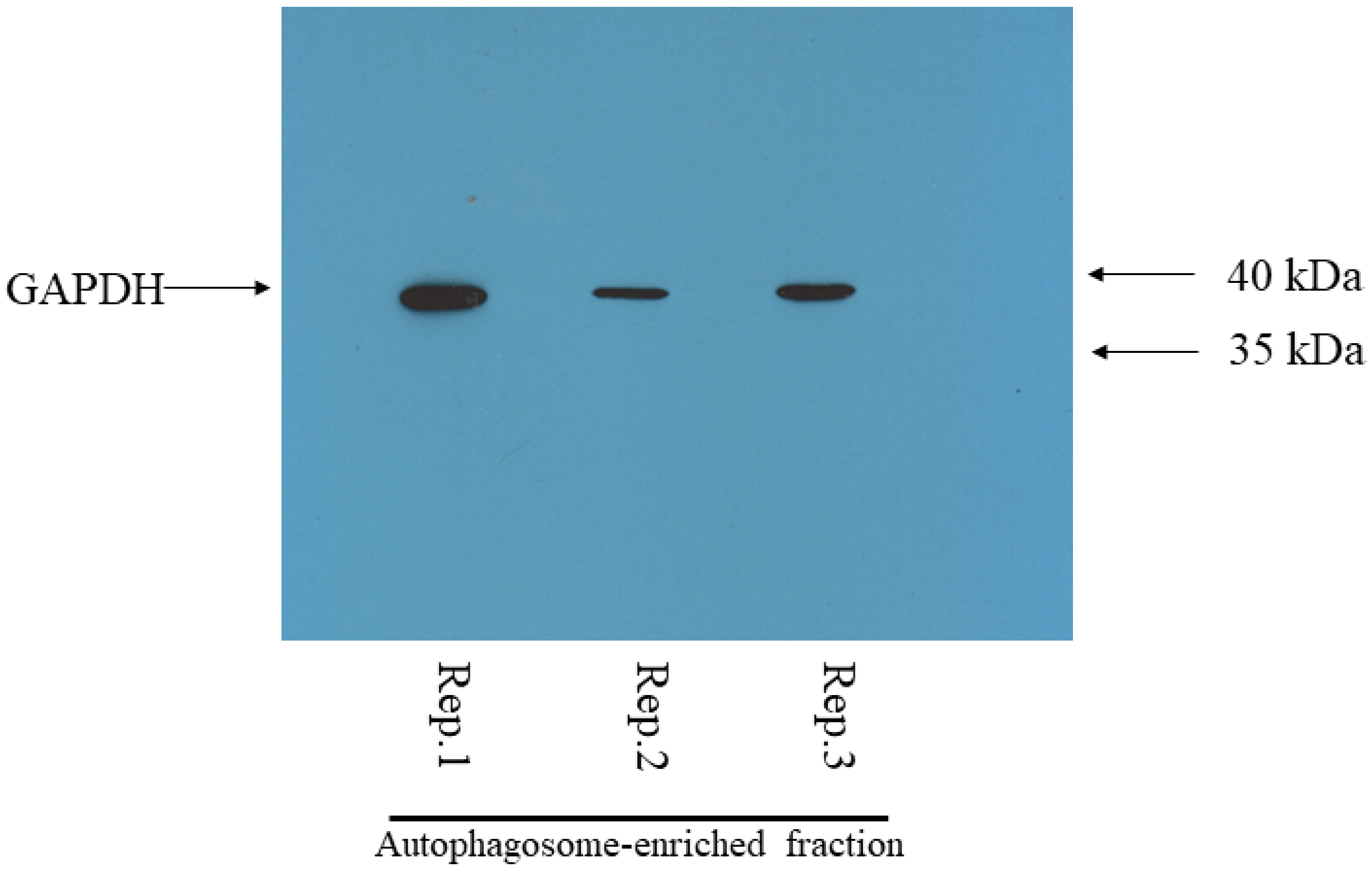

Image captured via ECL and X-ray film

Fig 2. Annexin-V Western Blot of autophagosome-enriched fractions from all three replicates

10% Tris-Glycine SDS-PAGE
Nitrocellulose

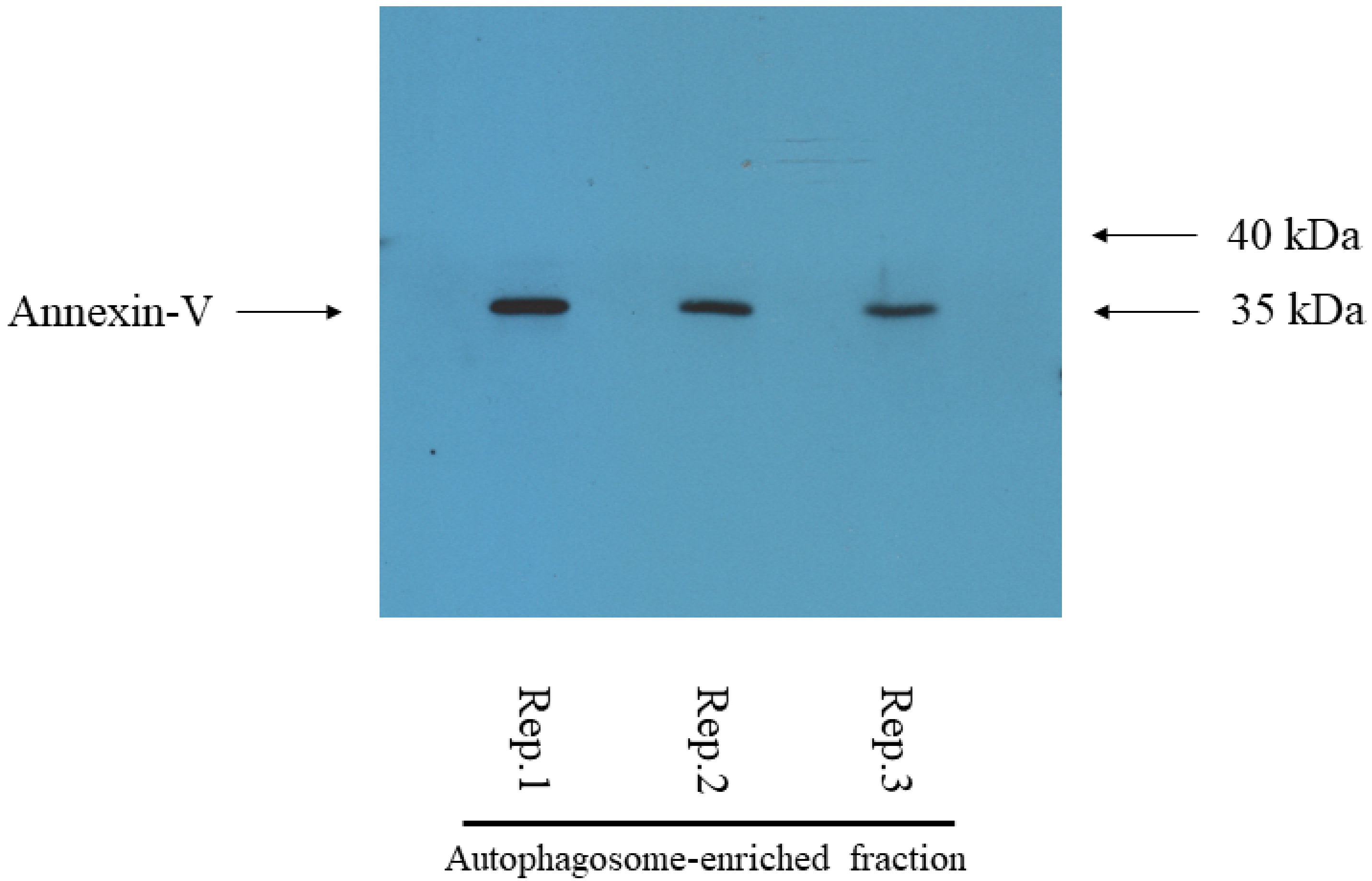

Image captured via ECL and X-ray film

Fig 2. *Ld*-EF1- α Dot Blot of autophagosome-enriched fractions from all three replicates

Dot Blot
Nitrocellulose

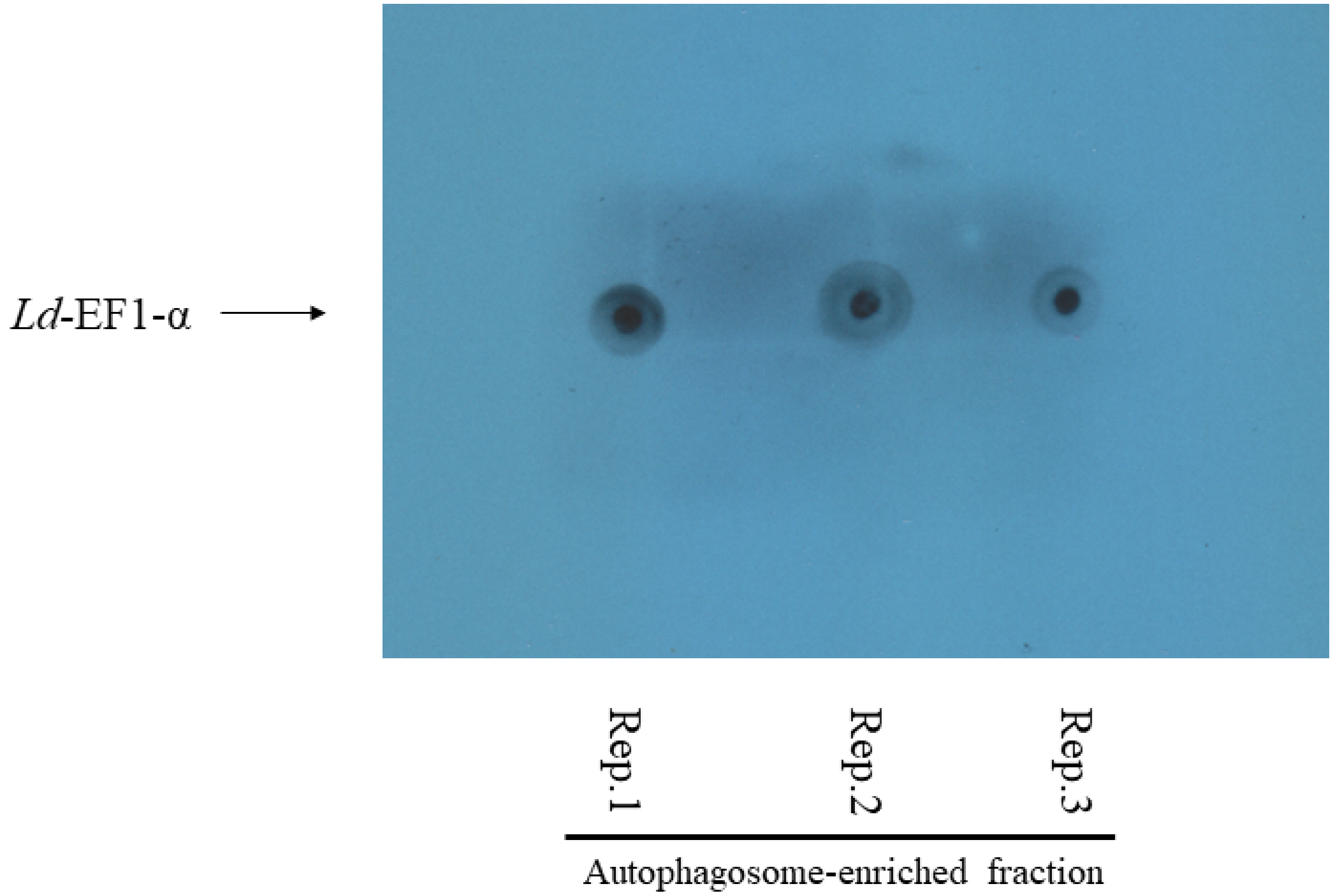

Image captured via ECL and X-ray film

Fig 2. *Ld*-aldolase Western Blot of autophagosome-enriched fractions from all three replicates

10% Tris-Glycine SDS-PAGE
Nitrocellulose

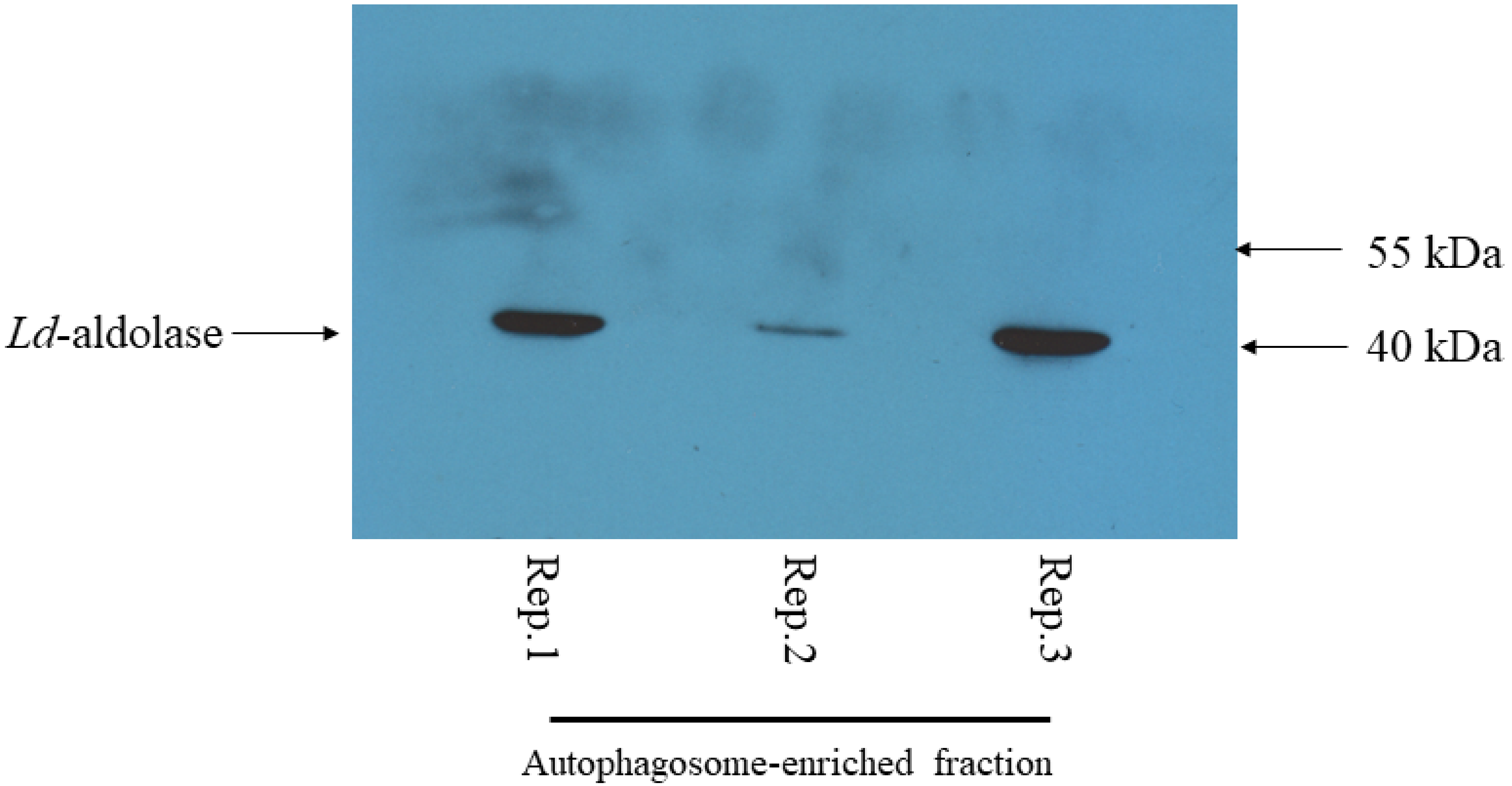

Image captured via ECL and X-ray film

Fig 2. Western blots for Lyn, GAPDH, Annexin-V, *Ld*-aldolase, and *Ld*-EF1-α in untreated dTHP-1 total cell lysate samples

10% Tris-Glycine SDS-PAGE
Nitrocellulose

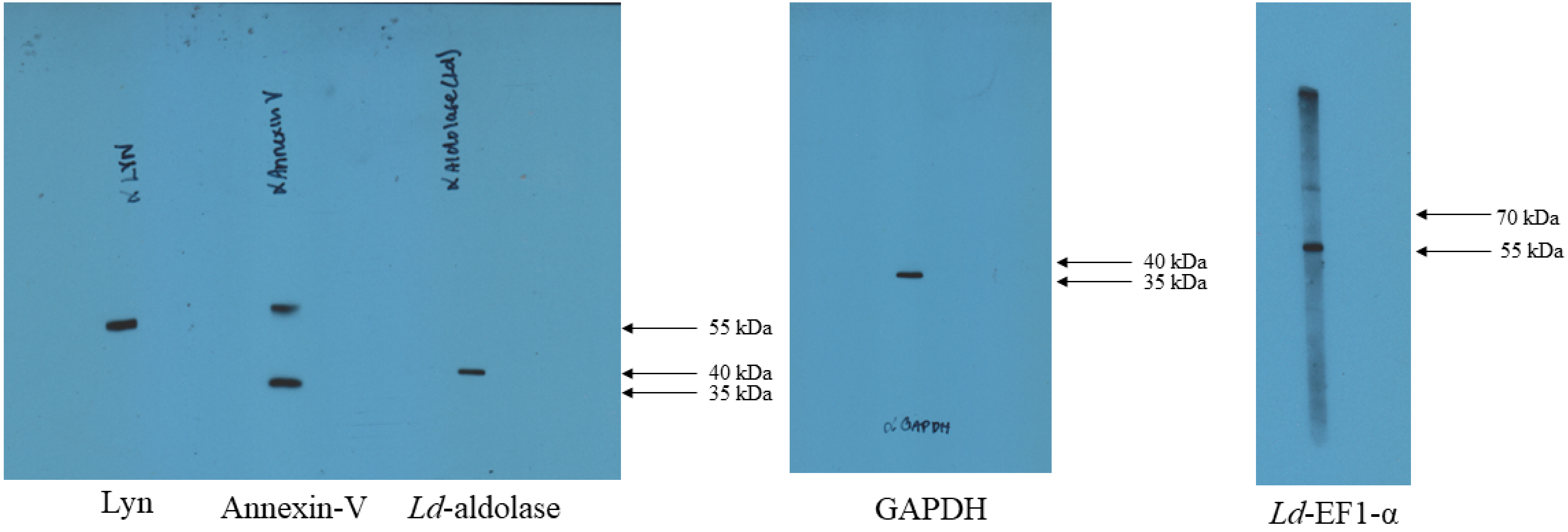

Images captured via ECL and X-ray film

Fig S2. LC3-II Western Blot of control and infected dTHP-1

15% Tris-Tricine SDS-PAGE
PVDF

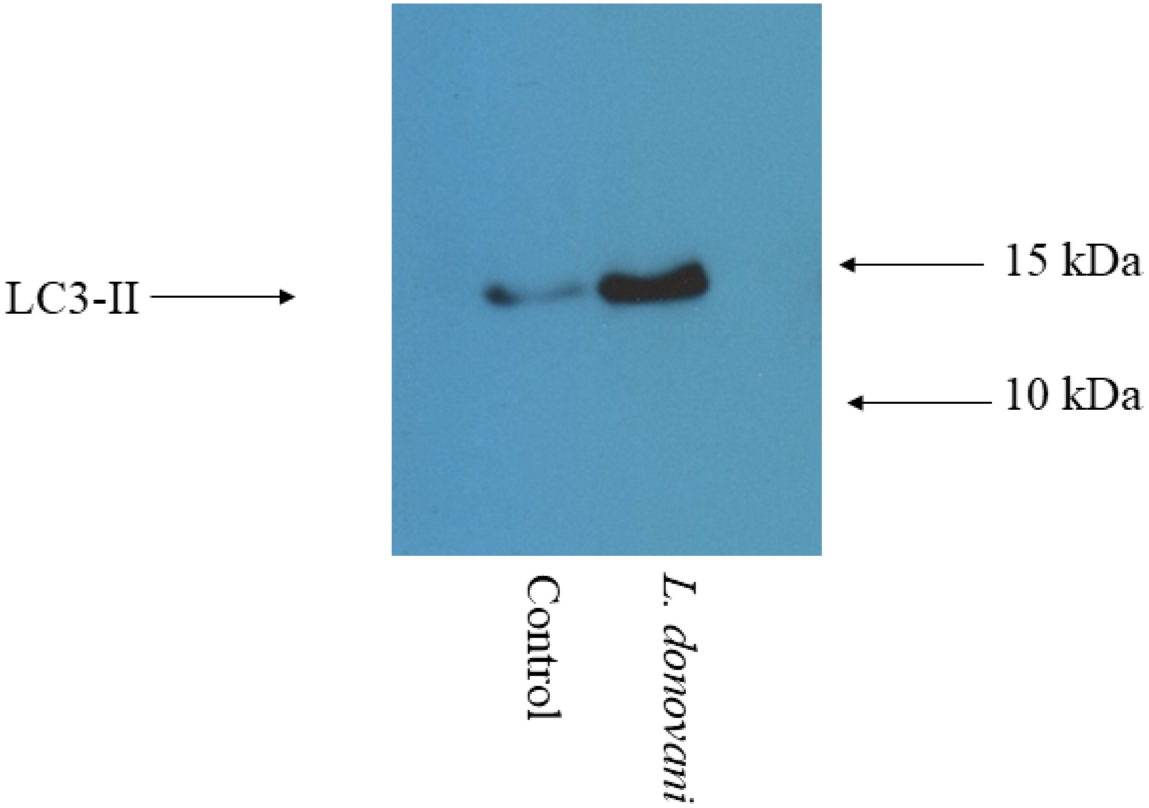

Image captured via ECL and X-ray film

Fig S2. β-actin Western Blot of control and infected dTHP-1

15% Tris-Tricine SDS-PAGE
PVDF

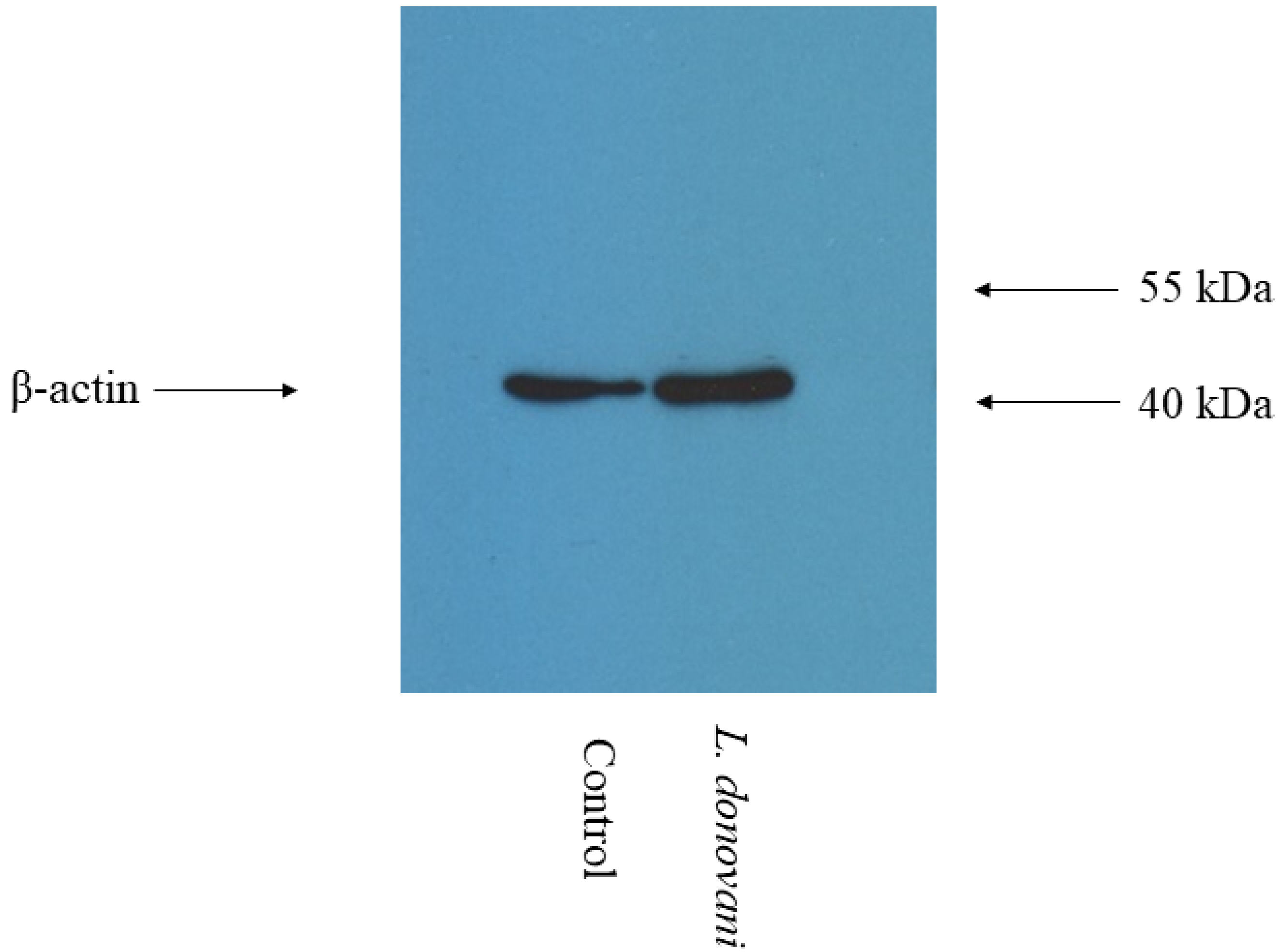

Image captured via ECL and X-ray film

Fig S2. LC3-II Western Blot of control and treated dTHP-1

15% Tris-Tricine SDS-PAGE
PVDF

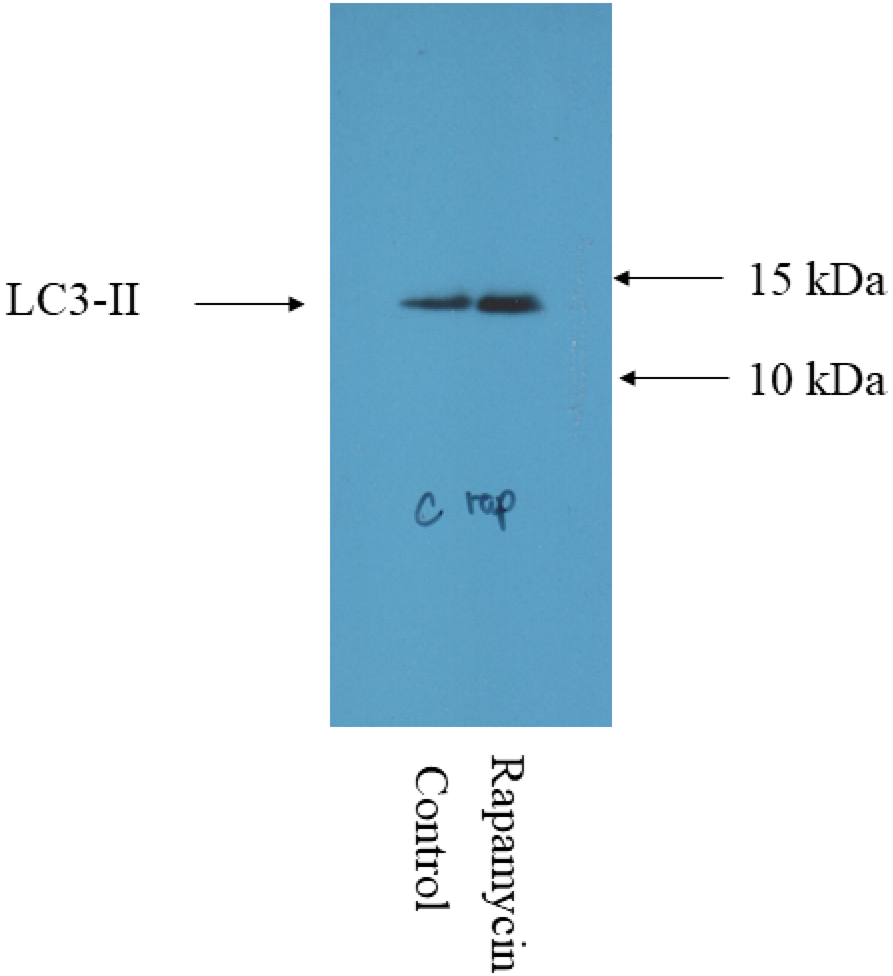

Image captured via ECL and X-ray film

Fig S2. β-actin Western Blot of control and treated dTHP-1

15% Tris-Tricine SDS-PAGE
PVDF

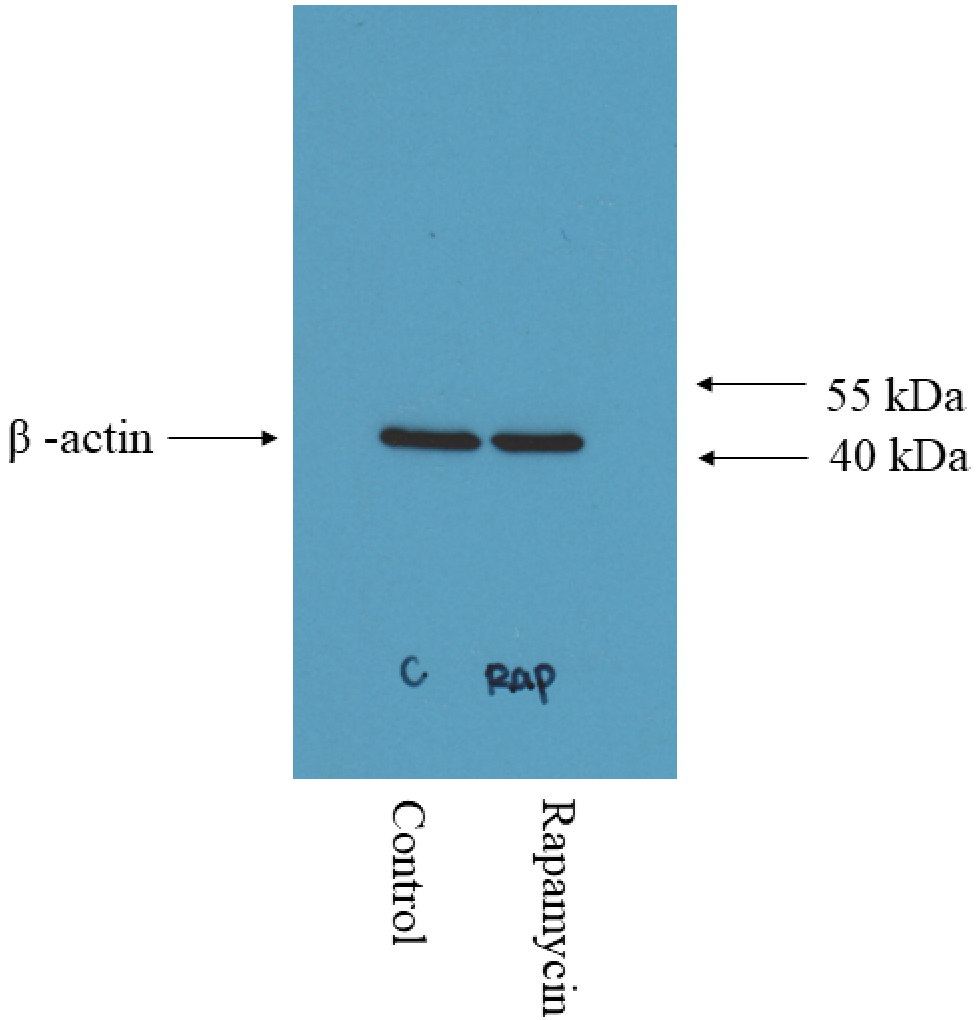

Image captured via ECL and X-ray film

Fig S2. LC3-II Western Blot of control and treated dTHP-1

15% Tris-Tricine SDS-PAGE
PVDF

LC3-II →

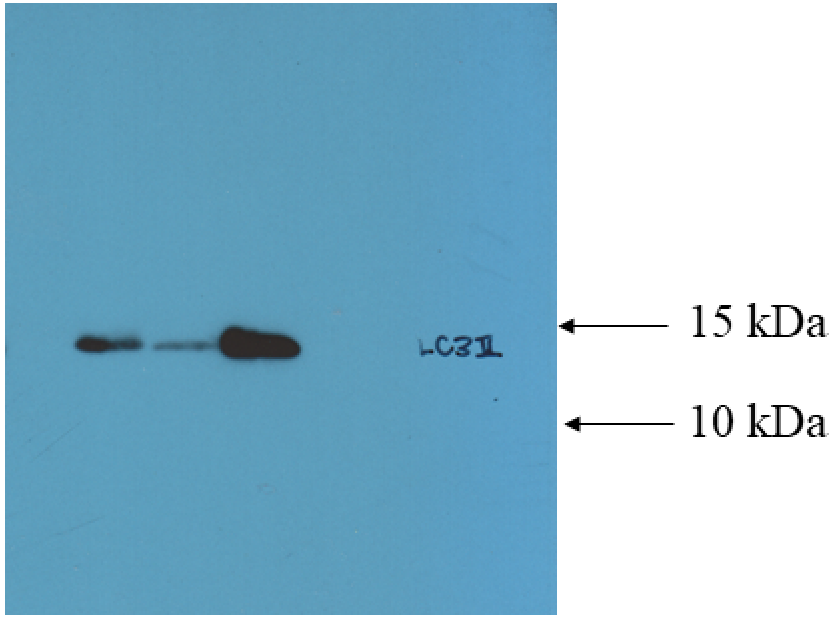

← 15 kDa

← 10 kDa

A B C

A. Control

B. Starvation

C. Starvation + Bafilomycin A1

Image captured via ECL and X-ray film

Fig S2. β-actin Western Blot of control and treated dTHP-1

15% Tris-Tricine SDS-PAGE
PVDF

B-actin →

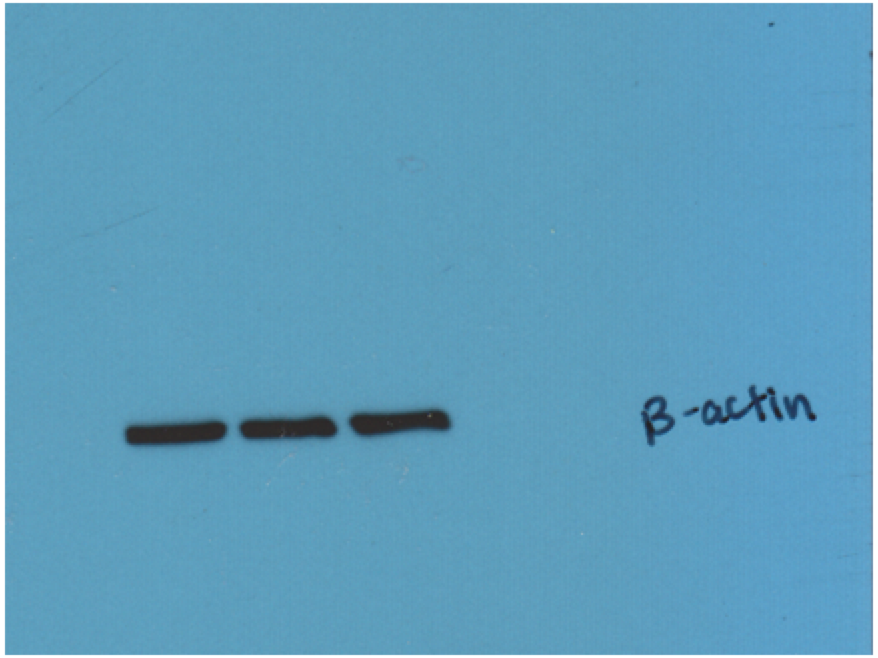

← 55 kDa
← 40 kDa

A B C

A. Control

B. Starvation

C. Starvation + Bafilomycin A1

Image captured via ECL and X-ray film

Fig S3. LC3-II Western Blot of control and treated dTHP-1

15% Tris-Tricine SDS-PAGE
PVDF

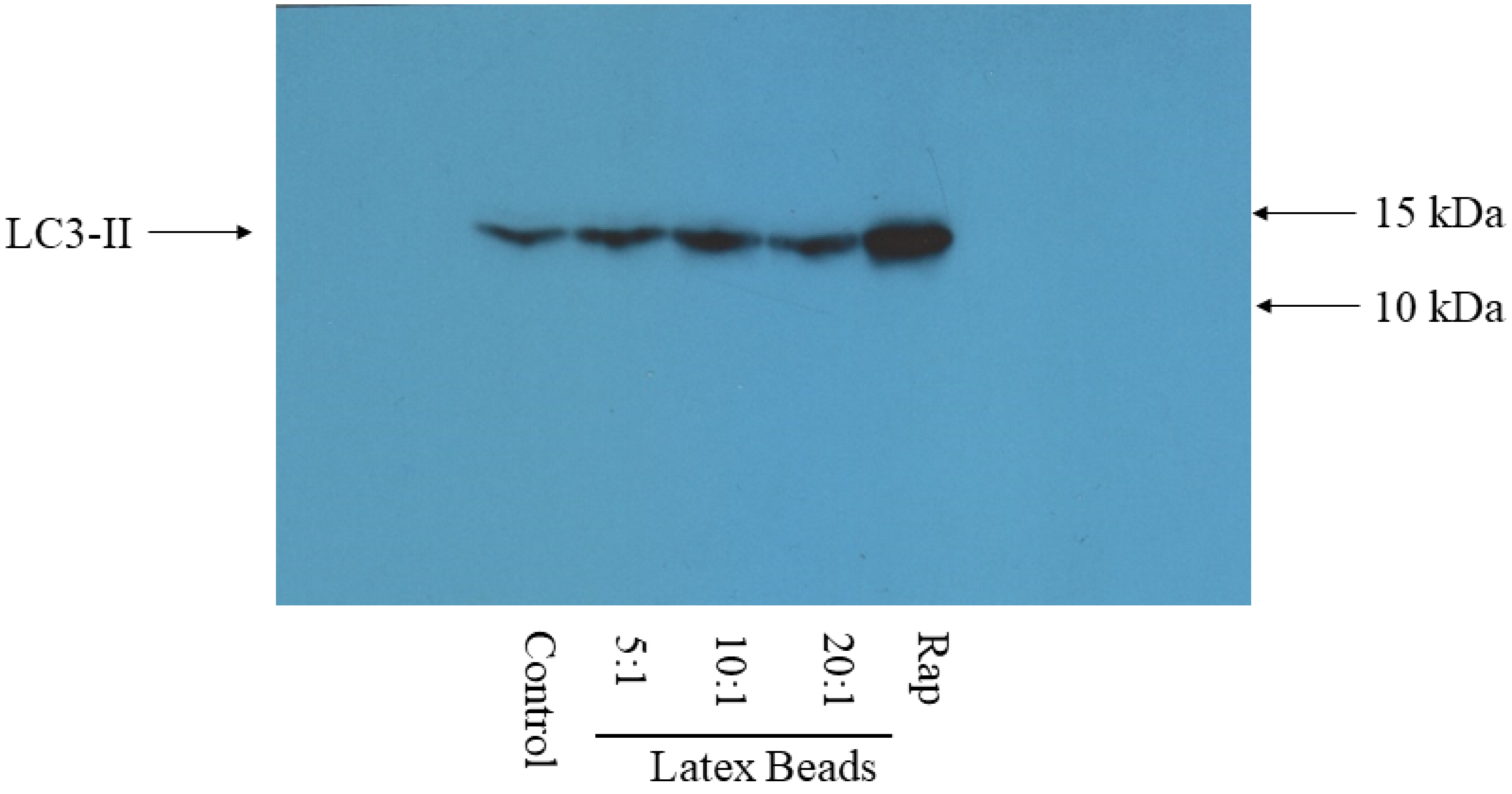

Image captured via ECL and X-ray film

Fig S3. β-actin Western Blot of control and treated dTHP-1

15% Tris-Tricine SDS-PAGE
PVDF

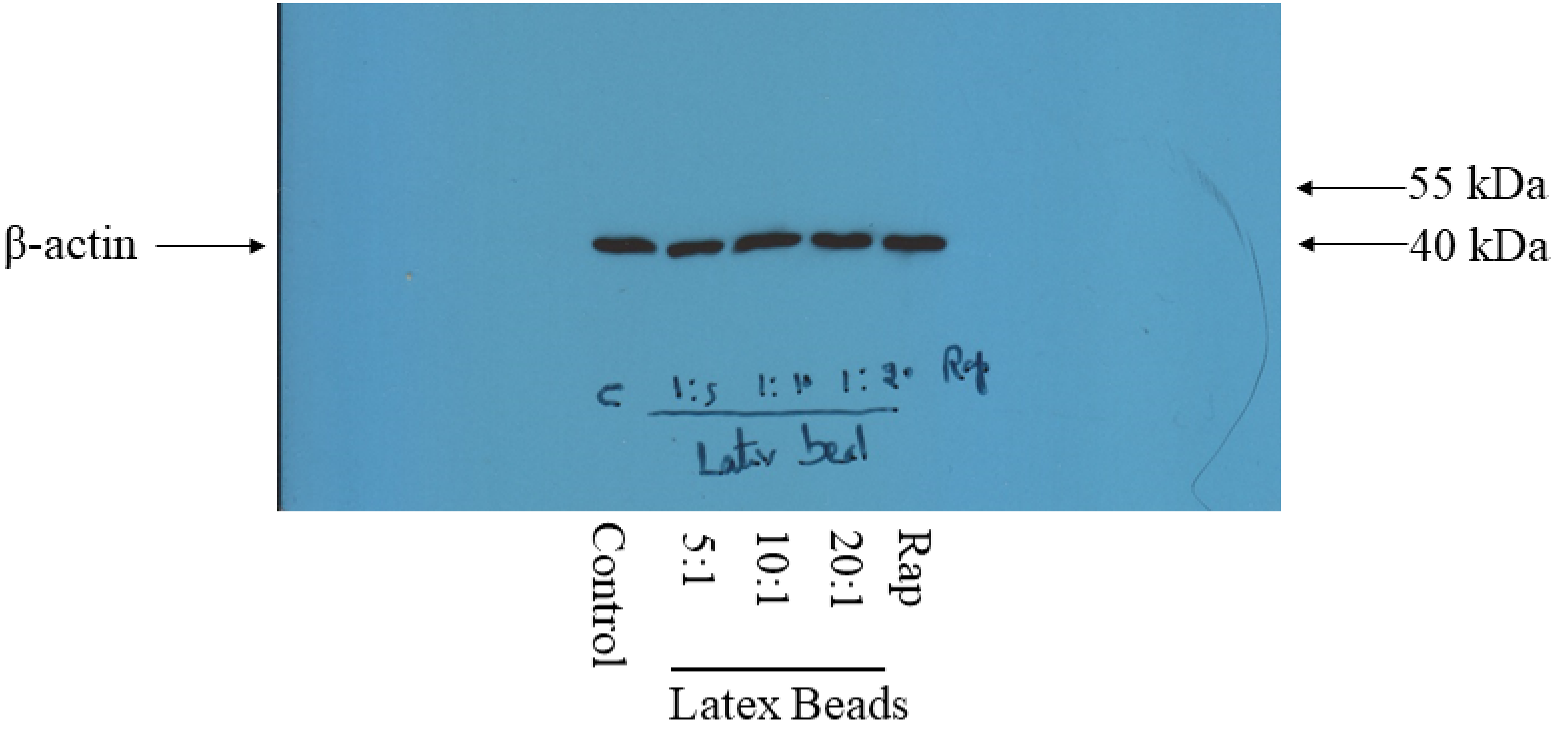

Image captured via ECL and X-ray film
